# Exploring the Role of Lipid-Binding Proteins and Oxidative Stress in Neurodegenerative Disorders: A Focus on the Neuroprotective Effects of Nutraceutical Supplementation and Physical Exercise

**DOI:** 10.3390/antiox11112116

**Published:** 2022-10-27

**Authors:** Giorgia Scarfò, Rebecca Piccarducci, Simona Daniele, Ferdinando Franzoni, Claudia Martini

**Affiliations:** 1 Department of Clinical and Experimental Medicine, Division of General Medicine, University of Pisa, 56126 Pisa, Italy; 2 Center for Rehabilitative Medicine “Sport and Anatomy”, University of Pisa, 56126 Pisa, Italy; 3 Department of Pharmacy, University of Pisa, 56126 Pisa, Italy

**Keywords:** lipid-binding proteins, neurodegenerative disease, apolipoproteins, nutraceutical supplement, physical activity

## Abstract

The human brain is primarily composed of lipids, and their homeostasis is crucial to carry on normal neuronal functions. In order to provide an adequate amount of lipid transport in and out of the central nervous system, organisms need a set of proteins able to bind them. Therefore, alterations in the structure or function of lipid-binding proteins negatively affect brain homeostasis, as well as increase inflammation and oxidative stress with the consequent risk of neurodegeneration. In this regard, lifestyle changes seem to be protective against neurodegenerative processes. Nutraceutical supplementation with antioxidant molecules has proven to be useful in proving cognitive functions. Additionally, regular physical activity seems to protect neuronal vitality and increases antioxidant defenses. The aim of the present review was to investigate mechanisms that link lipid-binding protein dysfunction and oxidative stress to cognitive decline, also underlining the neuroprotective effects of diet and exercise.

## 1. Introduction

Lipid homeostasis is a crucial mechanism for brain well-being, considering that lipids are the main components of neuronal cells and are also involved in bioenergetic and signaling pathways [1]. In order to ensure an adequate amount of lipids in and out of the Central Nervous System (CNS), human organisms require a set of proteins that are able to bind and transport lipids, called lipid-binding proteins (LBPs) [2]. The LBPs, also called lipid chaperones, are proteins able to bind lipids in a reversibly and non-covalently way [3]. Thus, the alterations of LBPs structure affect their functions impairing brain lipid homeostasis and exposing individuals to a high risk of developing neurodegenerative diseases (NDs) [1,4]. Indeed, LBPs are fundamental for organelle membranes, vesicle trafficking, myelin formation and degradation, misfolded protein accumulation, lysosome and proteasome function, as well as regulating inflammation and oxidative stress [2]. Among these molecular mechanisms influenced by LBPs, oxidative stress is for sure one of the main important processes contributing to NDs development [5]. In fact, free radicals lead to mitochondrial dysfunction, membrane instability, synapse impairment, apoptosis, and lipid peroxidation, which in turn favors autophagy, apoptosis, and ferroptosis contributing to cell death and loss of neurons [6,7,8].

In recent years, it has been demonstrated that environmental factors, including lifestyle, diet, and physical activity, affect the onset and development of NDs. Particularly, more and more attention has been focused on the neuroprotective effects of physical activity that could become an alternative way to act against oxidative stress and the consequent neurodegeneration. Particularly, exercise positively affects neuroplasticity and neuronal vitality, promoting angiogenesis, releasing neurotrophic factors and irisin, balancing autophagy and apoptosis, and reducing inflammation and oxidative stress [9].

Moreover, nutraceutical supplementation has been investigated since lots of antioxidant molecules (including vitamins C and E, polyphenols, and selenium) are considered potential additional treatments in NDs, even though their ability to pass the blood–brain barrier (BBB) and their effective bioavailability in the CNS is not yet fully clarified [10]. However, natural compounds can alter gut microbiota in a positive way, and the existence of a gut–brain axis regulates inflammation and oxidative stress in the CNS [11].

Overall, the aim of the present review was to investigate physiological, molecular mechanisms that link lipid metabolism dysregulation and lipid-binding protein dysfunction to NDs’ development, with particular attention on the neuroprotective role of nutraceutical supplements and physical activity.

## 2. Lipid-Binding Proteins in Neurodegenerative Disorders

Lipids are essential molecules for brain functions [12], and their management in the brain and across the BBB is strictly regulated by a set of non-enzymatic proteins, which are able to interact with them [2]. Among these, apolipoproteins are proteins capable of binding lipids, thus forming lipoproteins [13]. The lipids that constitute the apolipoproteins include cholesterol, triglycerides, and phospholipids, thus generating high-density and low-density lipoproteins (HDLs and LDLs) [14]. Different families of apolipoproteins exist as follows: Apo A (classified in Apo-A1, Apo-A2, Apo-A4, and Apo-A5), ApoB (classified in Apo-B48 and Apo-B100), ApoC (classified in Apo-C1, Apo-C2, Apo-C3, and Apo-C4), ApoD, ApoE, ApoF, ApoH, ApoL, and ApoM [15]. 

Among these, ApoA, ApoC, ApoE, and ApoJ were demonstrated to play a crucial role in the β-amyloid (Aβ) homeostasis, a neuropathological marker of Alzheimer’s disease (AD) included among the NDs, while ApoD is more generally essential in maintaining brain health.

Indeed, it was demonstrated that the deficiency of Apo-A1, which is the main component of HDLs, is able to favor β-amyloid (Aβ) deposition as well as its related inflammatory status and its oligomerization [16]. In turn, Aβ deposits affect their own clearance, directly impairing LDL receptor-related protein 1 (LRP1) in the hippocampus. In fact, LRP1 is responsible for transporting Aβ throughout the brain, and it proved to be oxidized by Aβ itself, which therefore worsens LPR1 functions [17]. Regarding ApoC, three different isoforms (Apo C-I, C-II, and C-III) were found abundant in the cerebrospinal fluid of patients genetically predisposed to develop AD, suggesting their involvement in the pathogenesis of AD [18]. The ApoJ is another LBP involved in Aβ pathology: it seems to be fundamental in the clearance of Aβ [19], although its exact role is controversial [20].

Notably, ApoD is involved in redox homeostasis within the brain since it is able to bind arachidonic acid and its derivatives, preventing their peroxidation [21]. Moreover, it would seem that the expression of ApoD is a homeostatic mechanism that the human brain implements in order to maintain oxidative stress and inflammation under control: in fact, ApoD-knockout mice are more predisposed to early brain aging, loss of neurons, cognitive decline, and neurological disorders [22]. Additionally, its antioxidant role, ApoD, is fundamental in regulating astrocyte vitality through autocrine mechanisms [23]. Furthermore, in murine models, it was demonstrated that ApoD is fundamental in myelin biogenesis since it provides the integrity of lysosomal membranes and a correct location of regulatory proteins, which are necessary for the compaction of myelin [24]. Supporting this, ApoD knockout mice show a decreased thickness of myelin with a consequent reduction in velocity in nerve conduction and a decline in cognitive and motor functions [25]. The correlation between lipid-binding proteins and myelin was investigated, given that myelin exerts a pivotal role in the conduction velocity, and its degeneration provokes a decline in cognitive functions [26].

In addition, in a drosophila model of the retina, some ApoD homolog lipocalins were proven to regulate the expression of numerous genes involved in autophagy mechanisms providing protection against type I spinocerebellar ataxia (SCA) degeneration [27]. Lipocalin 2 (Lcn2) is also involved in autophagy: outside the CNS, it was proven to enhance the permeability of lysosomal vesicles, impairing autophagy mechanisms and promoting cell death [28]. Through similar processes, Lcn2 probably mediates neuronal damages, particularly in hippocampal regions, as well as mediating microglial activation and neurotoxicity [29]. In contrast to ApoD involvement in myelin biogenesis, Lcn2 pathologically influences its catabolism. Moreover, a recent murine study showed that Lcn2-expressing astrocytes express phagocytic properties against myelin, leading to myelination [30]. In addition, Lcn2 was proven to be increased in multiple sclerosis (MS) brains and prevents remyelination processes [31].

In the field of lipid-binding proteins and their involvement in NDs development, ApoE is the main actor since it has been classified as one of the main risk factors for both early- and late-onset AD. Thus, a detailed description of this apolipoprotein is given in the following paragraph.

## 3. Apolipoprotein E (ApoE)

The ApoE is a protein mainly implicated in lipid homeostasis in both peripheral and central tissues; in particular, in the CNS, it modulates synaptic integrity/plasticity, glucose metabolism, and cerebrovascular functions [32]. ApoE is synthetized by several tissues, among which the liver, adipose tissue, endothelium, female reproductive system, and brain are the main ones; of note, about 75% of ApoE peripheral amount is attributed to liver production by hepatocytes and Kupffer cells [33]. The brain is the second most common site of ApoE production, and it is principally ascribed to glial cells, i.e., microglia, oligodendrocytes, and astrocytes, but also pericytes, vascular smooth muscle cells, choroid plexus, and neurons under stress conditions can produce it [34,35]. 

The involvement of ApoE in lipid homeostasis is prevalent. Indeed, ApoE is a lipid-binding protein that is implicated in lipid metabolism and transport through blood, lymph, cerebrospinal fluid, and interstitial fluid [35,36]. In particular, in plasma, ApoE is associated with chylomicron, very low-density lipoproteins (VLDLs), and HDLs. It mainly interacts with low-density lipoprotein receptors (LDLR) and their family members, such as LRP1, LRP8, and VLDL receptor (VLDLR) [32,36], as well as heparan sulfate proteoglycan (HSPG) receptors [37], thus promoting the clearance of plasma lipoproteins by endocytic pathway [38]. In the CNS, ApoE plays a key role in lipids and cholesterol transport [37]. Particularly, ApoE-mediated cholesterol exchange arises from non-neuronal (e.g., astrocytes) to neuronal cells and vice-versa (for instance, from neurons to microglia) [39], and it regulates lipid clearance and recycling [39]. Of note, the ApoE protein produced by peripheral tissues seems to not cross the BBB, underlining that the two pools, i.e., central and peripheral, are separate and distinct [35,40]. 

ApoE exists in three different isoforms, depending on the amino acid type present in position 112 and 158 of primary sequence: ApoE 2 (112Cys/158Cys), ApoE 3 (112Cys/158Arg), and ApoE 4 (112Arg/158Arg) [41]. Importantly, the single amino acid polymorphism contributes to ApoE structure and function alterations [32], resulting in changes in the protein conformation, post-translational modification, lipoprotein preference, and binding affinity for receptors [42]. The presence of ε4 allele is strongly associated with AD development, with a 9–15-fold increased risk for 4 homozygous compared to 4 heterozygous (3–4-fold increased risk) [43]. 

Beyond its role in lipid transport, ApoE is fundamental in Aβ production [44] and clearance [45]. Particularly, when ApoE binds to its own receptor, ERK1/2 MAP kinase pathways are activated and stimulate the transcription factor AP-1, which is responsible for increased transcription of amyloid-β precursor protein (APP), leading to enhanced production of Aβ; interestingly, the isoform ε4 is more efficient in triggering these pathways compared to ApoE ε2 and ApoE ε3 [44]. 

The ApoE ε4 effects on the brain are also macroscopically detected since it has proved to be related to hippocampal atrophy in subjects affected by mild cognitive impairment (MCI) [46], and the presence of the ApoE ε4 genotype seems to be linked to a faster decline in memory tests [47]. Moreover, the same isoform is also responsible for a rapid decline in language and executive functions in healthy individuals over 60 years old [48]. 

ApoE ε4 also negatively affects hippocampal neurogenesis, decreasing dendritic arborization and impairing memory functions [49]. In addition, ApoE ε4 polymorphism is associated with higher LDL cholesterol levels and a consequent enhanced risk of atherosclerosis, predisposing the ε4 carriers to cerebrovascular abnormalities and cognitive decline [50]. Moreover, ApoE ε4 is related to a worse prognosis after traumatic brain injuries (regardless of different types of damage) [51] as well as to other types of NDs, including Parkinson’s disease (PD) [52,53] and dementia with Lewy bodies (DLB) [54]. The mechanisms by which ApoE ε4 contributes to neurodegeneration development are numerous [55]. Apart from the above-mentioned involvement in a marked production of Aβ, the altered structure of the ε4 isoform surely affects the Aβ binding ability [56], thus impairing its transport to microglia [57] or through the BBB [58], thus altering its clearance [59]. ApoE ε4 also mediates the proteolytic degradation of Aβ less efficiently compared to ApoE ε2 and ApoE ε2 [60]. A recent study demonstrated that the selective removal of astrocytic ApoE ε4 seems to prevent tau hyperphosphorylation and tau-related neurodegeneration, suggesting that ApoE ε4 promotes neurotoxicity itself [61]. Furthermore, it was demonstrated that the allele ε4 negatively affects cholesterol homeostasis in the central nervous system predisposing to AD onset [62]. 

Of note, ApoE ε4 also negatively impacts synaptic plasticity. Specifically, it has been recently demonstrated that the allele ε4 is associated with deficits in extra-hippocampal memory and learning in young mice, and this is due to attenuated pre-synaptic plasticity in specific regions, thus suggesting that early modifications in ApoE ε4 brains could predispose to the AD development [63,64]. Notably, ApoE ε4 is also implicated in the alteration of neuroinflammation: particularly, ApoE ε4 microglia express increased tumor necrosis factor (TNF α) concentration than ε2 or ε3 microglial cells [65]. Similarly, human ApoE ε4 astrocytes show higher cytokine production, including interleukin 1β (IL-1β), TNFα, and IL-6, thus enhancing neuroinflammation and promoting AD development [66].

## 4. Fatty Acid Binding Proteins in Neurodegeneration

Fatty acid binding proteins (FABPs) are members of the LBPs family; they are intracellular molecules responsible for lipid transport inside cells [67] encoded by ten FABP genes in mammals. Three of them, FABP3, FABP5, and FABP7, are expressed in the brain and seem to be the effectors of several pathways that regulate microglia and brain development [68]. In particular, FABP3 is expressed in mature neurons, while FABP5 and FABP7 are mainly present in neural progenitor cells [69]. Although their primary structures show strong homology, they exhibit specific fatty acid ligand preferences. FABP3 binds preferentially to ω6 polyunsaturated fatty acid (PUFAs) such as arachidonic acid (20:4) (20–22), FABP5 prefers saturated fatty acids such as stearic acid (18:0) and monounsaturated fatty acids such as oleic acid (OA, 18:1) (22–24), and FABP7 binds preferentially to ω3 PUFAs such as docosahexaenoic acid (DHA, 22:6) (21,22,25) [69]. Notably, FABP3 could exert a crucial role in fetal neuronal development since it seems to be fundamental in ω3 and ω6 PUFA transport in mouse trophoblasts [70].

FABP3 expression has proven to correlate with the aggregation of α-synuclein (α-syn) in synucleinopathies, including PD [71]. α-syn is a 14 kDa protein encoded by the SNCA gene that is located on chromosome 4 [72], characterized by an amphipathic lysine-rich N-terminal domain capable of binding membrane lipids [73]. It seems that the expression of the SNCA gene is epigenetically influenced by aging and physical activity since it was demonstrated that healthy and active individuals had lower α-syn levels [74]. In physiological conditions, the exact role of α-syn is still questionable [75], but it seems to modulate synaptic vesicles acting on their mobilization and endocytosis [76]. In murine models, the suppression of FABP3 prevents the loss of dopaminergic neurons and both monomeric and fibrillar α-syn accumulation [77]. In fact, the interaction between FABP3 and α-syn is crucial since it negatively affects proteasome activity preventing α-syn degradation [78]. α-syn accumulation has proven to alter membrane permeability leading to an increased intracellular concentration of calcium, activation of calpain pathways, and synaptic dysregulation [79]. Additionally, α-syn impairs the polymerization of tubulin [80] and destabilizes microtubules, thus negatively affecting cytoskeleton functions [81]. In murine models, it was observed that the α-syn accumulation within the endoplasmic reticulum (ER) causes ER stress, which in turn enhances neurodegenerative processes in synucleinopathies [82]. Moreover, extracellular α-syn accumulation exerts neurotoxicity in PD models activating microglia with the involvement of several pathways triggered by the activation of the nuclear factor (NF)-κB, phosphatidylinositol 3 kinase (PI3K)/protein kinase B (AKT) and mitogen-activated protein kinases (MAPKs) [83,84]. Neurotoxicity is also mediated by the α-syn-induced release of inflammatory factors, including TNF-α, nitric oxide (NO), and interleukins, as well as reactive oxygen species (ROS) [85]. 

In addition to its role in PD, FABP3 is also considered a marker of AD because of its relation with Aβ accumulation [86]. It was even proposed that FABP3 negatively correlates with the severity of the disease: a large amount of FABP3 was detected in the CSF of AD brains, suggesting its correlation with neurodegenerative processes [87]. In this regard, considering that FABP3 is primarily found in gray matter, its location in the CSF is uncommon and reflects destructive processes that occur in neuronal membrane lipids [88]. Furthermore, a negative correlation between FABP3 and the Mini-Mental State Examination (MMSE) has also been recently demonstrated in the early stages of AD, underlining that FABP3 could be an early predictor of cognitive decline [87]. Similarly, FABP5 suppression in mice has been associated with memory impairment and cognitive decline [89]. In particular, FABP5 seems to affect learning and memory by activating PPARγ signaling pathways. Moreover, FABP5 was shown to regulate the transport through the BBB of the docosahexaenoic acid [90], which is a 22-carbon omega 3 PUFA with an essential role as a constituent of neuronal membranes [91]. Under oxidative stress conditions, FABP5 can bind α-syn and translocate to mitochondria, reducing membrane potential with the consequent impairment of mitochondrial activity [92]. In this regard, mitochondrial dysfunction is considered a primary cause of neurodegeneration in several synucleinopathies, suggesting a strict relation between α-syn and mitochondrial vitality [93]. In fact, α-syn influences several processes within mitochondria [94]: it impairs calcium signaling, thus altering its homeostasis [95]; favors mitochondrial fragmentation [96]; and alters their structure, therefore influencing the expression of proteins fundamental for mitochondrial morphology [97,98] α-syn overexpression also acts on the complex I of the electron transport chain impairing respiratory functions and leading to an increased genesis of free radicals [99]. 

In addition, in the CNS, FABP5 enhances malignancies of lower-grade gliomas since it induces the epithelial-mesenchymal transition triggering the TNFα/NF-κB pathway [100].

Beyond its role in neurons, FABP7 is a well-known modulator of astrocyte functions [101] by regulating synaptic functions and dendritic morphology [102]. In the amyotrophic lateral sclerosis (ALS) mice model, it was demonstrated that the overexpression of FAPB7 leads to astrocyte neurotoxicity, increasing the activity of the pro-inflammatory NF-κB pathway [103]. In addition, FABP7 seems to be upregulated by the Reelin-Dab1/Notch signal pathway, which is responsible for improving the migration of neuronal cells during brain development [68]. FABP7 is also an important regulator of PUFA levels in the brain: specifically, it was observed that the expression of FABP7 in malignant glioma cells was associated with an increased ω6/ω3 ratio, suggesting the involvement of FABP7 in the dysregulation of lipid homeostasis with deleterious effects on the brain [104].

Moreover, in AD brains of patients expressing ApoE ε4, there are reduced levels of FABP7, and this alters PUFA cerebral content and their role as anti-inflammatory molecules [105].

Although FABP3, FABP5, and FABP7 belong to the most represented LBPs in the CNS, it was also suggested that FABP4 could have a key role in neurodegeneration since it is expressed in mice microglial cells. In particular, FABP4 suppression leads to increased expression of mitochondrial uncoupling protein 2 (UCP2) with a consequent attenuation of the pro-inflammatory response mediated by palmitic acid, nitric oxide synthase (iNOS), and TNF-α [106]. 

When considering the above, the relationship among FABPs, inflammation, and oxidative stress suggests that these proteins are strongly involved in neurodegeneration and should be taken into account in order to improve the treatment of these diseases.

## 5. Cholesterol Synthesis and Metabolism in the CNS

Although hypercholesterolemia is known to be involved in neurodegenerative disorders, brain cholesterol is independent of circulating levels, and its metabolism in the CNS is crucial for neuronal health [107]. Specifically, brain cholesterol is produced by astrocytes, and its clearance depends on its conversion in 24(S)-hydroxycholesterol (24S-OHC) that is subsequently eliminated from the brain [108]. The efflux of brain cholesterol involves ApoE, Apo-A1, and ATP-binding cassette A1 (ABCA1); the latter is responsible for cholesterol transport from cells to poor-lipidated ApoA1 and ApoE [109]. 

However, the role of 24S-OHC in neurodegeneration is still unclear since it seems to both favor and prevent neuronal cell death [108]. Indeed, 24S-OHC is able to protect neurons from the cytotoxicity of 7-ketocholesterol, inducing their adaptations, as well as reducing Aβ accumulation and tau hyperphosphorylation. On the other hand, the same molecule seems to induce cell apoptosis favoring the activity of caspase proteins, leading to the formation of abnormal lipid structures, and increasing oxidative stress [110,111,112,113,114].

Oxidative stress and inflammation, in turn, negatively affect cholesterol homeostasis. The autoxidation of cholesterol forms oxysterols, including 24-hydroxycholesterol and 27-hydroxycholesterol, which were found to be higher in the cerebrospinal fluid of AD patients compared to controls. These oxysterols seem able to increase Aβ production besides being crucial in connection with the peripheral circulating cholesterol, considering that they can cross the BBB [110]. 

Cholesterol metabolism also seems to be involved in PD pathogenesis: cholesterol excess in lysosomes leads to abnormalities in the lipid rafts, which are membrane microdomains enriched in cholesterol and sphingolipids [111]. As a consequence, altered interactions between cholesterol and α-syn occur in brain cells, enhancing α-syn oligomerization and thus influencing PD pathogenesis [112]. Causes of this excess of cholesterol inside lysosomes could be mutations that occur in the GBA1 gene that encodes for the lysosomal enzyme β-glucocerebrosidase-1 and are shown to correlate with PD. Particularly, N370S-GBA1 mutation has proved to cause retention of β-glucocerebrosidase-1 in the endoplasmic reticulum altering lysosomal traffic and impairing autophagy with a consequent lysosomal cholesterol accumulation [113,114].

Finally, epigenetic mechanisms mediated by microRNAs (miRNAs) and affecting genes involved in cholesterol homeostasis seem to be crucial in neurodegeneration. It was suggested that miR-106b-5p, miR-758, and miR-33 downregulate the expression of the ABCA1 gene, with a consequent decrease in Apo-A1-related cholesterol efflux from the brain [109].

## 6. Oxidative Stress and Lipid Peroxidation

Oxidative stress is a condition that arises from an imbalance between the production of reactive oxygen species (ROS) and the antioxidant defenses. Generally, antioxidant defenses protect our cellular systems from possible oxidative damage, regulating ROS levels [115]. It was demonstrated that ROS play a useful role as signaling molecules to regulate cellular processes, including redox homeostasis [116,117,118]. Unfortunately, if ROS concentration is too high and/or the antioxidant defenses are lower to contrast them, this dysregulation causes oxidative stress [115,117]. 

Due to their unstable nature, ROS often react with cellular macromolecules (proteins, nucleic acid, and lipids), and these events are particularly evident in the brain [119]. Indeed, the brain is characterized by an elevated consumption of oxygen and glucose and has the highest rate of lipid metabolism in the body [120]. Moreover, neuronal cells are characterized by lowered enzymatic and nonenzymatic antioxidant mechanisms, impairing the neutralization of free radicals and thus enhancing oxidative stress [120]. This high metabolic rate triggers the production of free radicals and ROS, particularly in mitochondria, generated by the leakage of electrons out of the electron transport chain [121]. In contrast, cytosolic ROS derive from the NADPH oxidase (NOX) and, depending on the conditions, from other enzymes, including xanthine oxidase [122,123].

The cell membranes are highly susceptible to ROS damage since they are rich in lipids, especially PUFAs, thus going towards lipid peroxidation, which is particularly involved in NDs [124]. Indeed, lipid peroxidation generates a large number of free radicals triggering neuronal membrane damage and producing secondary products, which contribute to extensive cellular damage [119]. As mentioned, lipid peroxidation targets preferentially PUFAs, including linoleic acid, arachidonic acid (AA), and docosahexaenoic acid (DHA), which are highly consumed by the brain, thus corroborating the high level of lipid turnover in this organ [119]. 

Lipid peroxidation is a non-enzymatic process and involves distinct steps of initiation, propagation, and termination [125]. In the first stage, reactive oxygen metabolites form a fatty acid radical by reacting with the carbon–carbon double bond of PUFAs. These compounds are unstable and are inclined to form conjugated dienes, generating carbon-centered alkyl radicals [126]. In the propagation step, the subsequent generation of a lipid peroxyl radical leads to the attack of an additional PUFA molecule, alimenting an uncontrolled self-perpetuating chain reaction in which every PUFA of the cellular membrane can potentially be oxidized [127]. Finally, the termination is guaranteed by substrate depletion, the generation of stable products derived from the mutual reaction of all the present radicals, and the presence of a molecule with antioxidant and chain-breaking properties [124].

ROS production not only leads to phospholipids damage but also attacks membrane proteins and induces lipid-protein and protein-protein crosslinking, thus altering membrane integrity [128]. The lipid peroxidation carries to membrane permeability alteration together with a decreased membrane fluidity, lower membrane-bound enzyme activity, and membrane receptors impairment [129,130].

Not surprisingly, the above-mentioned alterations and modifications of lipids have been demonstrated to affect neuronal homeostasis and contribute to brain dysfunction. A huge amount of data clearly demonstrate that ROS and oxidative stress have a pivotal role in the development of NDs [131,132,133].

In particular, several papers reported that brain tissues and body fluids of patients affected by NDs, including AD, PD, ALS, Huntington’s disease (HD), and Down syndrome (DS) [134,135,136,137,138], present higher levels of lipid peroxidation markers with respect to control subjects. The alteration in lipid peroxidation was demonstrated to be accompanied by an elevation of ROS in the brain areas associated with the specific neurodegenerative phenomenon [135,139,140].

Moreover, the process of lipid peroxidation involves several signaling cascades, including the activation of phospholipases and their subsequent physiological responses [121,139].

Beyond the cell membrane, even mitochondria membranes are particularly sensitive to lipid peroxidation that contributes to mitochondrial dysfunction [120]. Since mitochondria are the main source of free radicals that derive from the electron transport chain, these organelles are susceptible to ROS damage and ROS-induced mutations [140]. It was demonstrated that the oxidation of cardiolipin, a glycerophospholipid that constitutes the mitochondrial inner membrane, leads to an increased membrane permeability favoring access to pro-apoptotic molecules, including BAX and BAD, which reduce mitochondria survival [141]. Mitochondria play a crucial role in NDs; indeed, a recent study showed that abnormalities that occur in these organelles are responsible for cell loss in the pedunculopontine nucleus in PD brains [142]. Moreover, an altered mitochondrial cytochrome oxidase gene expression was proven to be associated with AD [143]. In addition, lipid peroxidation in AD murine models is directly involved in the process of amyloidogenesis since it is able to upregulate the expression of BACE1 (beta-site amyloid precursor protein cleavage enzyme 1) and the consequent increased beta-secretase activity and Aβ production [144]. 

**Figure 1:**
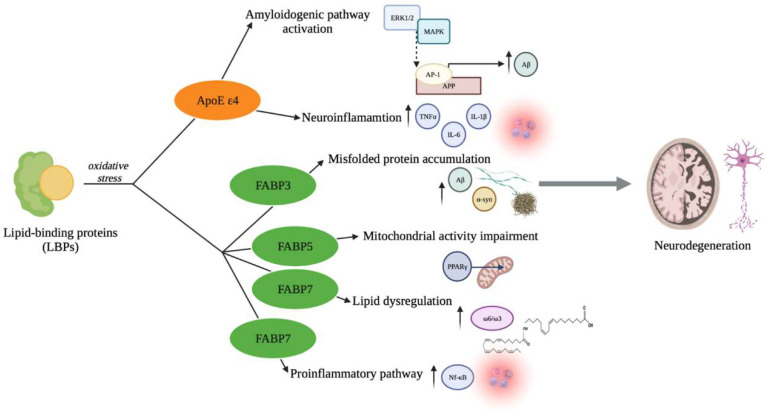
Lipid-binding proteins and oxidative stress in the pathogenesis of neurodegeneration. Oxidative stress is one of the main processes that modulate the LBPs functions. Indeed, when the production of ROS is predominant compared to antioxidant defenses activation, the apolipoproteins and FABPs activity are altered. Under these conditions, the ApoE ε4 mainly modulates the amyloidogenic process activation by the ERK1/2 MAP kinase pathway that induces the Aβ production in relation to AP-1-dependent APP transcription, and neuroinflammation, especially by promoting TNF-a, IL-1β, and IL-6 release. At the same time, FABP3 induces Aβ and a-syn accumulation and correlates with the alterations of cognitive performance, thus being considered an early predictor of cognitive decline. Similarly, under oxidative stress, FABP5 binds a-syn and affects the PPAR_γ_ pathway, thus leading to mitochondria dysfunctions. Moreover, FABP7 promotes lipid dysregulation by increasing the ω6/ω3 ratio, as well as induces the proinflammatory pathway activity, promoting NF-kB release. Overall, these processes contribute to defining the pathological mechanisms that conduct the neurodegeneration onset. The figure was created in BioRender.com, accessed on 18 September 2022.

The crucial involvement of LPBs and oxidative stress in neurodegeneration development is schematically represented in Figure 1.

## 7. Antioxidant Supplementation in Neurodegenerative Diseases

**Figure 2:**
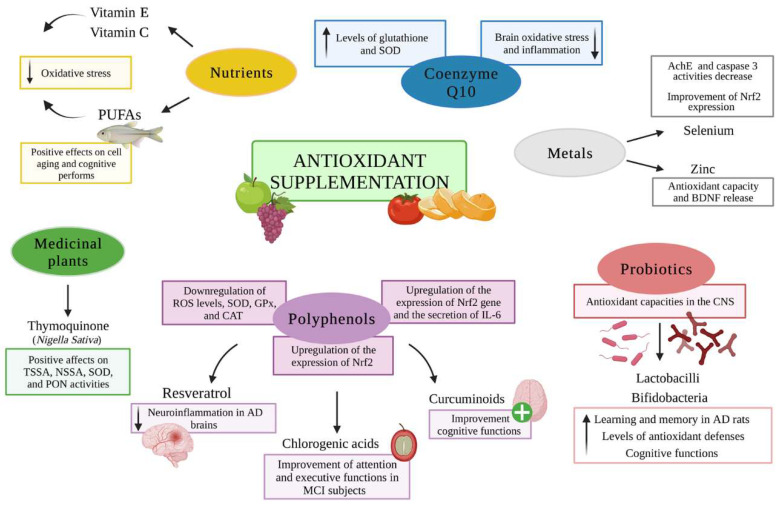
Antioxidant supplementation in neurodegenerative diseases. Antioxidant supplementations exhibit beneficial effects on the onset and progression of neurodegenerative diseases. Vitamins E and C play an important role in reducing oxidative stress, while PUFAs exert positive effects on cell aging and cognitive performance. Coenzyme Q10 (2 weeks) is able to decrease brain oxidative stress and inflammation, improving levels of glutathione and superoxide dismutase (SOD). Regarding metals, zinc was demonstrated to improve cognitive functions (antioxidant capacity and BDNF release), while selenium regulates oxidative stress status and apoptosis (activities of AChE and caspase 3 decrease). Polyphenols can downregulate ROS levels, SOD, GPx, and CAT, and upregulate the expression of Nrf2, enhancing attention, executive (chlorogenic acids), and cognitive functions (curcuminoids) as well as reducing neuroinflammation in the AD brains (resveratrol). Active compounds in medicinal plants, such as thymoquinone, can counteract oxidative stress in the brains of rats exposed to head irradiation, affecting TSSA, NSSA, SOD, and PON activities. Probiotics exhibit antioxidant activity in CNS, improving some manifestations of neurodegenerative disorders. The figure was created in BioRender.com, accessed on 18 September 2022.

Antioxidants exert the main role in oxidative chain reaction termination through the elimination of free radical intermediates [145]. In particular, the use of antioxidant compounds has been elected as a candidate approach to prevent or counteract neuronal cell death; moreover, supplementation of the diet with nutraceutical products has been proposed to contribute to limiting the pathology progression in early phases of NDs [146]. Indeed, thanks to their well-known antioxidant properties, many natural compounds play a crucial role in delaying the onset and the progression of neurodegenerative disorders [10], as represented in Figure 2. 

In this regard, it was demonstrated that vitamins C and E are effective against oxidative stress; the co-supplementation with 400 IU of vitamin E and 1000 mg of vitamin C in AD patients is able to pass through the BBB, and elevated levels of vitamins were found in the CSF; this reflected in a decreased lipoprotein peroxidation [147]. 

PUFAs are also useful antioxidant molecules; in particular, the ω3 PUFA has proved to improve both the oxidative stress status and inflammation since it is able to reduce circulating levels of cytokines with a notable positive effect on cognitive perform [148]. Additionally, ω3 PUFA positively affected cell aging, influencing telomere length [148]. Moreover, the PUFA ω3, such as eicosapentaenoic acid (EPA) and docosahexaenoic acid (DHA), whose principal dietary sources are fatty cold-water fishes, induce the fasting and postprandial serum triglyceride (TG) by reducing VLDL hepatic synthesis without hepatic retention of lipids [149,150,151]. Phytosterols (nuts, seeds, and vegetable oils) promote LDL-cholesterol (LDL-C) decrease in a dose-dependent way [152]. In particular, phytosterols do not allow intestinal cholesterol absorption increasing LDL receptors and, at the same time, inducing LDL-C concentrations to decrease [153,154].

A 2-week coenzyme Q10 (CoQ10) supplementation, a component of the inner mitochondrial membrane [155], was proven effective in reducing brain oxidative stress and inflammation by its influence on mitochondrial dysfunction. In particular, it seems to improve levels of glutathione and superoxide dismutase (SOD) [156]. In this regard, the CoQ10 deficiency was demonstrated to be a biomarker of oxidative stress in PD [157]. 

Concerning polyphenols, resveratrol, presents in red grapes, peanuts, and other plants, is able to attenuate neuroinflammation in AD brains since it is an activator of the SIRT1 pathway [158], regulating redox homeostasis [159]. Chlorogenic acids (CGAs), polyphenols that are contained in coffee beans, have been shown to improve attention and executive functions in subjects suffering from MCI [160]. Similarly, in AD murine models, CGAs were demonstrated effective in modulating oxidative stress through the inhibition of acetylcholinesterase (AChE) and butyrylcholinesterase (BChE), which are two enzymes with a known pro-oxidant activity [161]. Among polyphenols, curcuminoids were proven to attenuate oxidative stress, affecting the activity of SOD, glutathione peroxidase (GPx), and catalase (CAT), and prevent lipid peroxidation and apoptosis in neuronal cells, thus improving cognitive functions [162]. More generally, most of the polyphenols are able to downregulate ROS levels, SOD, GPx, and CAT and upregulate the expression of nuclear factor erythroid 2-related factor 2 (Nrf2) that encodes antioxidant enzymes; additionally, the polyphenols act on inflammation regulating the expression of nuclear factor kappa-light-chain-enhancer of activated B cells (NF-κB) gene and the secretion of IL-6 [163]. In addition, Polyphenols limit lipid peroxidation and oxidative stress of plasma lipoproteins as well as promote hepatic LDL receptors [164]. Moreover, dietary polyphenols intake could guarantee the improvement of HDL-C levels [165], and isoflavones such as genistein (e.g., soy) are potent antioxidants against LDL oxidation [166]. 

Among the medicinal plants, Nigella Sativa and its active compound, thymoquinone, are highly effective on oxidative stress in the brains of rats exposed to head irradiation since they positively affect the total superoxide scavenger activity (TSSA), non-enzymatic superoxide scavenger activity (NSSA), SOD, and paraoxonase (PON) activities [167].

Furthermore, among metals, the effects of zinc supplementation on brain functions were also studied: it seems to be able to improve cognitive functions, enhancing the release of BDNF and the antioxidant capacity [168]. In murine models, instead, the supplementation with selenium nanoparticles decreases the activity of AChE and caspase 3 and improves the expression of Nrf2, thus regulating the oxidative stress status and apoptosis [169]. It was demonstrated that a zinc deficiency, a nutritional problem that may lead to atherosclerosis onset, could decrease hepatic Apo-A1 gene expression and reduce plasma Apo-A1 levels [170].

Finally, probiotics, a group of non-pathogenic microorganisms, exert antioxidant capacities in the CNS [10] and improve manifestations of several neurodegenerative diseases, including PD and AD [171]. It was demonstrated that Lactobacilli and Bifidobacteria improve learning and memory in AD rats [172]. In another murine model, the supplementation with Lactobacilli-fermented cow’s milk increases levels of antioxidant defenses (SOD, GSH, and GP), reduces inflammatory factors, and improves cognitive functions both in vivo and in vitro [173]. In AD patients, a 12-week consumption of probiotic-treated milk improved oxidative stress and inflammation, reducing plasma malondialdehyde (MDA), a lipid peroxidation end product, and serum high-sensitivity C-reactive protein (hs-CRP) with a clear positive effect on the MMSE [174]. A possible mechanism through which probiotics act on the antioxidant defenses in the CNS is the activation of the SIRT1 pathway, as demonstrated by Bonfili et al. in mice treated with a probiotic formulation supplementation (Slab51) [175]. In fact, SIRT1, an NAD^+^-dependent protein deacetylase, is known as a promoter of cell survival thanks to its ability to lower ROS levels in the CNS [176]. 

In PD subjects, a randomized, double-blind, placebo-controlled trial demonstrated that a 12-week Lactobacillus supplementation improves the patient score on the Unified Parkinson’s Disease Rating Scale (UPDRS) and glutathione (GSH) levels as well as decreased levels of hs-CRP and MDA [177]. Probiotics in PD were also proven to act on the expression of inflammation-related genes with downregulation of IL-1, IL-8, and TNF-α and upregulation of TGF-β and PPAR-γ [178]. 

## 8. Neuroprotective Effects of Physical Activity

**Figure 3:**
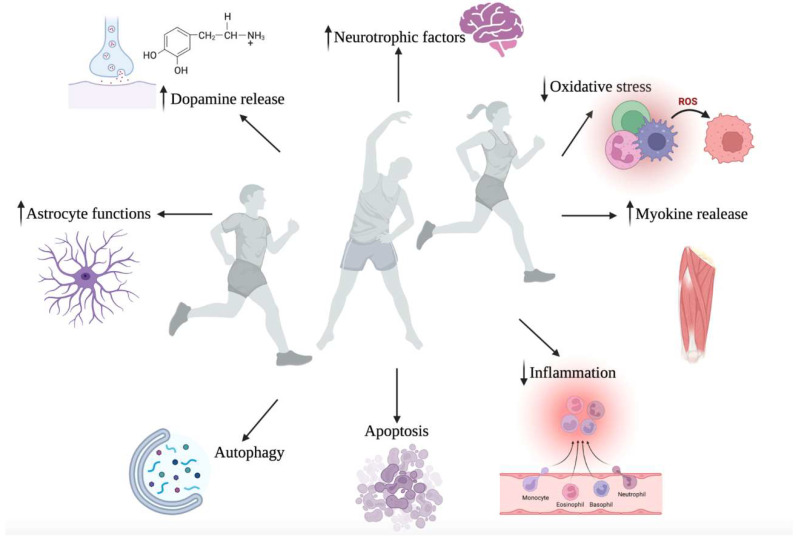
Neuroprotective effects of physical activity. Regular exercise is demonstrated to decrease oxidative stress and inflammation as well as increase the release of anti-inflammatory molecules, including IL-4 and IL-10. In addition, exercise triggers the PGC-1α/FNDC5 pathway in skeletal muscles, with the consequent release of Irisin, which is fundamental for the increase in neurotrophic factors. PA improves astrocyte functions with positive effects on hippocampal and vascular activities. In addition, exercise seems able to restore the AMPK and the JNK-BCL2 autophagy, preventing the accumulation of misfolded proteins and ensuring mitochondrial turnover. Exercise also acts on cell apoptosis and brain aging, restoring Wnt/β-Catenin signaling. Finally, PA enhances the release of dopamine improving brain functions in patients suffering from PD. The figure was created in BioRender.com, accessed on 18 September 2022.

Beyond the neuroprotective effects of nutraceutical supplementation, the beneficial role of physical activity (PA) in protecting from NDs development is growing [179] (Figure 3). Physical activity has well-known neuroprotective effects, improving neurotrophic factors production, synaptic connections, and neuronal survival [9]. Exercise has also been proven to be related to morphological changes in the CNS, affecting cognitive functions positively. First, it was demonstrated that aerobic training is able to enhance hippocampal perfusion, which is reflected in its enlargement with a remarkable improvement in memory [180]. In another study, it was shown that a 12-week exercise intervention was able to ameliorate the functional connectivity of the hippocampus in subjects suffering from MCI [181]. This effect could be mediated by the release of neurotrophic factors, including brain-derived neurotrophic factor (BDNF), nerve growth factor (NGF), and insulin growth factor -1 (IGF-1); both strength and endurance interventions are useful in increasing BDNF and IGF1 in old adults [182]. In fact, BDNF, with its autocrine and paracrine functions, acts on glutamatergic and GABAergic synapses as well as serotonergic and dopaminergic transmission with clear positive effects on neuroplasticity [183]. BDNF is also crucial for the development of the postnatal hippocampus since it influences dendritic spine morphology, which is fundamental for memory [184]. Moreover, in the aging hippocampus of murine models, swimming exercise has been shown to increase IGF-1 triggering the IGF1/PI3K/Akt and AMPK/SIRT1/PGC1α and thus suppressing inflammation and apoptosis in neuronal cells [185]. 

The presence of muscle-brain crosstalk is well documented. Skeletal muscle produces myokines, which are molecules capable of regulating brain functions [186], and BDNF production seems to be dependent on FNDC5, which is an exercise-induced muscle protein [187]. Particularly, by triggering the PGC-1α/FNDC5 pathway in skeletal muscles, PA promotes BDNF increase in hippocampal tissues [187]. In addition, FNDC5 is also the precursor of irisin (another myokine) that has proved to be able to mediate the positive effects of physical activity on aging brains [188]. Specifically, irisin acts both on neuroplasticity and synaptic health, favoring the release of neurotrophic factors (including BDNF) [188] and on acute brain injury: indeed, in a post-stroke brain, the ischemic damage usually activates the NOD-like receptor pyrin 3 (NLRP3) inflammasome that is responsible for mediating the inflammatory response, and it was demonstrated that irisin alleviates neuronal injuries inhibiting the NLRP3 inflammatory pathway [189]. 

The impact of PA on inflammation, which is crucial in neurodegenerative disorders, is supported by exercise capacity for modulating cytokine release: different kinds of training were proven to decrease IL-6 and TNFα levels [190,191] as well as intercellular adhesion molecule (ICAM-1), vascular cell adhesion molecule (VCAM-1), and E-selectin, reducing endothelial activation among elderly population [192].

Moreover, both resistance and endurance training favor the release of SIRT1, an NAD^+^-dependent protein deacetylase that plays a remarkable role in modulating inflammation and microglia activation [193]. SIRT1 mediates the PGC-1α deacetylation, activating several pathways (through the nuclear respiratory factors, Nrf1 and 2) that regulate mitochondria biogenesis and respiratory function, thus reducing inflammation and oxidative stress [194].

In murine AD models, a 4-week treadmill exercise intervention was demonstrated to be efficient on memory functions, and this effect was mediated by the increase in anti-inflammatory cytokines IL-4 and IL-10, a decrease in IL-1β and TNF-α, peroxynitrite production, and lipid peroxidation. Furthermore, in the same study, PA preserved neuronal vitality through the suppression of the caspase-9/caspase-3 apoptotic pathway suggesting that exercise is particularly effective against inflammation and oxidative stress damage [195]. However, it has to be considered that regular moderate exercise has been widely demonstrated to counteract oxidative stress-related negative changes. In contrast, acute and vigorous exercise has been related to an excess of free radical production [132,196].

At rest, oxidative stress status is generally found to be lower in athletes than in sedentary individuals [197]. Therefore, the literature data present conflicting results due to differences in exercise intensities and duration as well as different methods used to estimate oxidative stress status. Conversely, antioxidant enzymes, including CAT and GPx, were reported to be increased in athletes than in sedentary individuals, although these changes are actually related to the physical status and training level of the athletes [198,199]. In this regard, PA was also proven to be efficient in reducing lipid peroxidation by the modulation of MDA and the activity of several enzymes, including SOD, CAT, GPx, and AChE [200,201].

Moreover, in neurodegenerative disorders, inflammatory status derives also from astrocyte activation that provides for negative changes in brain tissue [202]: in AD mice, exercise affects the astrocyte state increasing levels of glial fibrillary acid protein (GFAP) with a consequent enhancement of astrocytic BDNF and better hippocampal functions [203]. In addition, moderate levels of PA in rats induce astrocytes to cover brain vessels, restoring a better microcirculatory status that is often altered in neurodegenerative disease-affected brains [204].

The importance of regular physical activity in relation to neurodegenerative disorders also lies in the regulation of brain autophagy: impaired autophagy is known to be associated with misfolded protein accumulation, and exercise seems to be able to restore this mechanism by upregulating miR-130a and thus activating AMPK-mediated autophagy [205]. In addition, exercise induces mitophagy through the activation of AMPK and JNK-BCL2 pathways, and this process is fundamental for an adequate mitochondrial turnover in neuronal cells [206]. Exercise also acts on cell apoptosis and brain aging, restoring Wnt/β-Catenin signaling: in fact, the suppression of Wnt-associated pathways causes synaptotoxicity, neuronal apoptosis, and cognitive decline [207].

Exercise also plays a crucial role in delaying the progression of PD, acting directly on dopamine circuits [208]: in particular, aerobic exercise has been proven to enhance the release of dopamine in the caudate, simultaneously improving the sensibility of the ventral striatum affecting the dopaminergic pathway in a positive way [209]. 

Finally, in murine models, it was demonstrated that exercise reduces stress hormones, including corticosterone circulating levels, with remarkable positive effects on memory functions [200].

## 9. Conclusions

Lipid homeostasis is a crucial factor for brain well-being since lipids are fundamental in regulating brain functions. The alteration of LBPs and, consequently, the lipid contents in brain cells leads to NDs development, especially triggered by an impaired balance between ROS production and antioxidant defenses. The knowledge of neuroprotective effects given by nutraceutical supplements as well as physical activity is growing in the scientific community. Antioxidants exert a crucial role in modulating oxidative stress and inflammation, limiting the production of free radicals and increasing antioxidant defenses delaying brain aging and neurodegeneration. In addition, nutraceuticals positively affect lipid profile and lipoproteins, thus preventing also all mechanisms related to vascular impairment. These bioactive compounds do not substitute pharmacological therapies in severe diseases cases, but they could be used as possible adjuvants based on their potential effects on human health [210,211]. However, the activity of natural compounds has been mainly tested on animal models and by in vitro assays [211], while pharmaceutical compounds are produced based on good manufactured practices (GMPs), including a widespread series of preclinical and clinical studies and assuring high safety levels [212]. Furthermore, nutraceuticals do not need the approval of health authorities, unlike pharmaceuticals. Therefore, nutraceutical safety is not guaranteed, and, in this regard, it is possible that these products may also have undesirable effects [212]. As their consumption is becoming more common, people should keep more attention, and, as reported by Cicero et al. (2018), supplements should be recommended by professional sanitary figures to limit improper use [210]. Although many of these natural compounds are provided by dietary sources [213], they can be taken as nutraceutical supplementations to improve their bioavailability and potential beneficial effects based on higher concentrations. Nevertheless, it is important to underline that these supplements do not replace a balanced diet [214].

Furthermore, regular and moderate exercise is fundamental in improving antioxidant defenses and modulating oxidative stress without forgetting that physical activity favors the release of neurotrophic factors and stimulates skeletal muscles to produce myokines, which directly affect brain functions. Some studies confirmed that physical activity, particularly aerobic exercise, can modify the lipid profile, improving human health and counteracting, for example, dyslipidemias or nonalcoholic fatty liver disease (NAFLD), without side effects [215]. However, more studies are needed to better investigate the beneficial effects of exercise and nutraceuticals on lipid-binding proteins.

**Figure 4:**
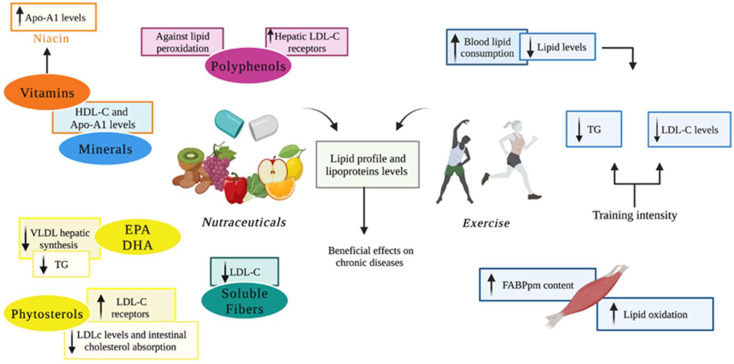
Beneficial effects of nutraceuticals and exercise on lipid profile. Nutraceuticals and exercise are able to influence lipid profile and lipoproteins levels. A good lifestyle improves health and prevents possible chronic diseases. Nutraceuticals are provided by dietary sources, but they generally contain higher concentrations of bioactive compounds, exerting potential beneficial effects. The ω3 EPA and DHA were demonstrated to reduce VLDL hepatic synthesis and serum TG, while phytosterols impede intestinal cholesterol absorption, increasing LDL-C receptors and reducing LDL-C levels. Micronutrients are involved in regulating some apolipoproteins gene expression and affect HDL-C metabolism. Minerals such as magnesium and zinc and vitamins such as ascorbic acid and niacin impact lipoproteins and Apo-A1 levels. Polyphenols exert antioxidant activity against lipid peroxidation and promote hepatic LDL receptors. Soluble fibers encourage LDL-C decrease. In parallel, physical exercise promotes blood lipid consumption, reducing their levels, and induces lipid oxidation in skeletal muscle, giving the energy required during training. Moreover, in skeletal muscle, physical activity increases FABPpm content, especially in males. Exercise training induces LDL reduction and TG levels, especially when exercise intensity grows. The figure was created in BioRender.com, accessed on 18 September 2022.

Herein, differently from the previous literature reviews in this field, we explored the interplay among lipids, lipid-binding proteins, and oxidative stress in order to offer an overview of lipid-related mechanisms involved in neurodegeneration onset, with particular attention to the antioxidant and neuroprotective role of nutraceutical supplement and physical activity as a non-pharmacological way of intervention (Figure 4).

## References

[1] Kao Y.-C., Ho P.-C., Tu Y.-K., Jou I.-M., Tsai K.-J. (2020). Lipids and Alzheimer’s Disease. IJMS.

[2] Corraliza-Gomez M., Sanchez D., Ganfornina M.D. (2019). Lipid-Binding Proteins in Brain Health and Disease. Front. Neurol..

[3] Glatz J.F.C. (2015). Lipids and Lipid Binding Proteins: A Perfect Match. Prostaglandins Leukot. Essent. Fat. Acids.

[4] Dey M., Gunn-Moore F.J., Platt B., Smith T.K. (2020). Brain Region–Specific Lipid Alterations in the PLB4 HBACE1 Knock-in Mouse Model of Alzheimer’s Disease. Lipids Health Dis..

[5] Losada-Barreiro S., Bravo-Díaz C. (2017). Free Radicals and Polyphenols: The Redox Chemistry of Neurodegenerative Diseases. Eur. J. Med. Chem..

[6] Salim S. (2017). Oxidative Stress and the Central Nervous System. J. Pharmacol. Exp. Ther..

[7] Kamat P.K., Kalani A., Rai S., Swarnkar S., Tota S., Nath C., Tyagi N. (2016). Mechanism of Oxidative Stress and Synapse Dysfunction in the Pathogenesis of Alzheimer’s Disease: Understanding the Therapeutics Strategies. Mol. Neurobiol..

[8] Su L.-J., Zhang J.-H., Gomez H., Murugan R., Hong X., Xu D., Jiang F., Peng Z.-Y. (2019). Reactive Oxygen Species-Induced Lipid Peroxidation in Apoptosis, Autophagy, and Ferroptosis. Oxid. Med. Cell. Longev..

[9] Mahalakshmi B., Maurya N., Lee S.-D., Bharath Kumar V. (2020). Possible Neuroprotective Mechanisms of Physical Exercise in Neurodegeneration. IJMS.

[10] Franzoni F., Scarfò G., Guidotti S., Fusi J., Asomov M., Pruneti C. (2021). Oxidative Stress and Cognitive Decline: The Neuroprotective Role of Natural Antioxidants. Front. Neurosci..

[11] Agirman G., Yu K.B., Hsiao E.Y. (2021). Signaling Inflammation across the Gut-Brain Axis. Science.

[12] Montesinos J., Guardia-Laguarta C., Area-Gomez E. (2020). The Fat Brain. Curr. Opin. Clin. Nutr. Metab. Care.

[13] Ramasamy I. (2014). Recent Advances in Physiological Lipoprotein Metabolism. Clin. Chem. Lab. Med. (CCLM).

[14] Mahley R.W., Innerarity T.L., Rall S.C., Weisgraber K.H. (1984). Plasma Lipoproteins: Apolipoprotein Structure and Function. J. Lipid Res..

[15] Liu T., Chen J.-M., Zhang D., Zhang Q., Peng B., Xu L., Tang H. (2021). ApoPred: Identification of Apolipoproteins and Their Subfamilies With Multifarious Features. Front. Cell Dev. Biol..

[16] Button E.B., Boyce G.K., Wilkinson A., Stukas S., Hayat A., Fan J., Wadsworth B.J., Robert J., Martens K.M., Wellington C.L. (2019). ApoA-I Deficiency Increases Cortical Amyloid Deposition, Cerebral Amyloid Angiopathy, Cortical and Hippocampal Astrogliosis, and Amyloid-Associated Astrocyte Reactivity in APP/PS1 Mice. Alz. Res. Ther..

[17] Owen J.B., Sultana R., Aluise C.D., Erickson M.A., Price T.O., Bu G., Banks W.A., Butterfield D.A. (2010). Oxidative Modification to LDL Receptor-Related Protein 1 in Hippocampus from Subjects with Alzheimer Disease: Implications for Aβ Accumulation in AD Brain. Free. Radic. Biol. Med..

[18] Hu Y., Meuret C., Martinez A., Yassine H.N., Nedelkov D. (2021). Distinct Patterns of Apolipoprotein C-I, C-II, and C-III Isoforms Are Associated with Markers of Alzheimer’s Disease. J. Lipid Res..

[19] Zandl-Lang M., Fanaee-Danesh E., Sun Y., Albrecher N.M., Gali C.C., Čančar I., Kober A., Tam-Amersdorfer C., Stracke A., Storck S.M. (2018). Regulatory Effects of Simvastatin and ApoJ on APP Processing and Amyloid-β Clearance in Blood-Brain Barrier Endothelial Cells. Biochim. Biophys. Acta (BBA)-Mol. Cell. Biol. Lipids.

[20] Yerbury J.J., Poon S., Meehan S., Thompson B., Kumita J.R., Dobson C.M., Wilson M.R. (2007). The Extracellular Chaperone Clusterin Influences Amyloid Formation and Toxicity by Interacting with Prefibrillar Structures. FASEB J..

[21] Dassati S., Waldner A., Schweigreiter R. (2014). Apolipoprotein D Takes Center Stage in the Stress Response of the Aging and Degenerative Brain. Neurobiol. Aging.

[22] Sanchez D., Bajo-Grañeras R., Del Caño-Espinel M., Garcia-Centeno R., Garcia-Mateo N., Pascua-Maestro R., Ganfornina M.D. (2015). Aging without Apolipoprotein D: Molecular and Cellular Modifications in the Hippocampus and Cortex. Exp. Gerontol..

[23] Bajo-Grañeras R., Ganfornina M.D., Martín-Tejedor E., Sanchez D. (2011). Apolipoprotein D Mediates Autocrine Protection of Astrocytes and Controls Their Reactivity Level, Contributing to the Functional Maintenance of Paraquat-Challenged Dopaminergic Systems. Glia.

[24] García-Mateo N., Pascua-Maestro R., Pérez-Castellanos A., Lillo C., Sanchez D., Ganfornina M.D. (2018). Myelin Extracellular Leaflet Compaction Requires Apolipoprotein D Membrane Management to Optimize Lysosomal-Dependent Recycling and Glycocalyx Removal. Glia.

[25] Ganfornina M.D., Do Carmo S., Martínez E., Tolivia J., Navarro A., Rassart E., Sanchez D. (2010). ApoD, a Glia-Derived Apolipoprotein, Is Required for Peripheral Nerve Functional Integrity and a Timely Response to Injury: Glial ApoD in Nerve Function and Regeneration. Glia.

[26] Ohno N., Ikenaka K. (2019). Axonal and Neuronal Degeneration in Myelin Diseases. Neurosci. Res..

[27] del Caño-Espinel M., Acebes J.R., Sanchez D., Ganfornina M.D. (2015). Lazarillo-Related Lipocalins Confer Long-Term Protection against Type I Spinocerebellar Ataxia Degeneration Contributing to Optimize Selective Autophagy. Mol. Neurodegener..

[28] Sung H.K., Chan Y.K., Han M., Jahng J.W.S., Song E., Danielson E., Berger T., Mak T.W., Sweeney G. (2017). Lipocalin-2 (NGAL) Attenuates Autophagy to Exacerbate Cardiac Apoptosis Induced by Myocardial Ischemia: Lipocalin-2, autophagy and cell death. J. Cell. Physiol..

[29] Kim J.-H., Ko P.-W., Lee H.-W., Jeong J.-Y., Lee M.-G., Kim J.-H., Lee W.-H., Yu R., Oh W.-J., Suk K. (2017). Astrocyte-Derived Lipocalin-2 Mediates Hippocampal Damage and Cognitive Deficits in Experimental Models of Vascular Dementia: KIM et Al. Glia.

[30] Wan T., Zhu W., Zhao Y., Zhang X., Ye R., Zuo M., Xu P., Huang Z., Zhang C., Xie Y. (2022). Astrocytic Phagocytosis Contributes to Demyelination after Focal Cortical Ischemia in Mice. Nat. Commun..

[31] Al Nimer F., Elliott C., Bergman J., Khademi M., Dring A.M., Aeinehband S., Bergenheim T., Romme Christensen J., Sellebjerg F., Svenningsson A. (2016). Lipocalin-2 Is Increased in Progressive Multiple Sclerosis and Inhibits Remyelination. Neurol. Neuroimmunol. Neuroinflam..

[32] Yamazaki Y., Zhao N., Caulfield T.R., Liu C.-C., Bu G. (2019). Apolipoprotein E and Alzheimer Disease: Pathobiology and Targeting Strategies. Nat. Rev. Neurol..

[33] Martínez-Martínez A.B., Torres-Perez E., Devanney N., Del Moral R., Johnson L.A., Arbones-Mainar J.M. (2020). Beyond the CNS: The Many Peripheral Roles of APOE. Neurobiol. Dis..

[34] Zhang Y., Chen K., Sloan S.A., Bennett M.L., Scholze A.R., O’Keeffe S., Phatnani H.P., Guarnieri P., Caneda C., Ruderisch N. (2014). An RNA-Sequencing Transcriptome and Splicing Database of Glia, Neurons, and Vascular Cells of the Cerebral Cortex. J. Neurosci..

[35] Chernick D., Ortiz-Valle S., Jeong A., Qu W., Li L. (2019). Peripheral versus Central Nervous System APOE in Alzheimer’s Disease: Interplay across the Blood-Brain Barrier. Neurosci. Lett..

[36] Huang Y., Mahley R.W. (2014). Apolipoprotein E: Structure and Function in Lipid Metabolism, Neurobiology, and Alzheimer’s Diseases. Neurobiol. Dis..

[37] Raffaï R.L., Hasty A.H., Wang Y., Mettler S.E., Sanan D.A., Linton M.F., Fazio S., Weisgraber K.H. (2003). Hepatocyte-Derived ApoE Is More Effective than Non-Hepatocyte-Derived ApoE in Remnant Lipoprotein Clearance. J. Biol. Chem..

[38] Getz G.S., Reardon C.A. (2009). Apoprotein E as a Lipid Transport and Signaling Protein in the Blood, Liver, and Artery Wall. J. Lipid Res..

[39] de Chaves E.P., Narayanaswami V., Christoffersen C., Nielsen L.B. (2008). Apolipoprotein E and Cholesterol in Aging and Disease in the Brain. Future Lipidol..

[40] Linton M.F., Gish R., Hubl S.T., Bütler E., Esquivel C., Bry W.I., Boyles J.K., Wardell M.R., Young S.G. (1991). Phenotypes of Apolipoprotein B and Apolipoprotein E after Liver Transplantation. J. Clin. Investig..

[41] Emi M. (1988). Genotyping and Sequence Analysis of Apolipoprotein E Isoforms*1. Genomics.

[42] Kanekiyo T., Xu H., Bu G. (2014). ApoE and Aβ in Alzheimer’s Disease: Accidental Encounters or Partners?. Neuron.

[43] Neu S.C., Pa J., Kukull W., Beekly D., Kuzma A., Gangadharan P., Wang L.-S., Romero K., Arneric S.P., Redolfi A. (2017). Apolipoprotein E Genotype and Sex Risk Factors for Alzheimer Disease: A Meta-Analysis. JAMA Neurol..

[44] Huang Y.-W.A., Zhou B., Wernig M., Südhof T.C. (2017). ApoE2, ApoE3, and ApoE4 Differentially Stimulate APP Transcription and Aβ Secretion. Cell.

[45] Piccarducci R., Daniele S., Polini B., Carpi S., Chico L., Fusi J., Baldacci F., Siciliano G., Bonuccelli U., Nieri P. (2021). Apolipoprotein E Polymorphism and Oxidative Stress in Human Peripheral Blood Cells: Can Physical Activity Reactivate the Proteasome System through Epigenetic Mechanisms?. Oxidative Med. Cell. Longev..

[46] Fleisher A. (2005). Sex, Apolipoprotein E Ε4 Status, and Hippocampal Volume in Mild Cognitive Impairment. Arch. Neurol..

[47] Caselli R.J., Reiman E.M., Locke D.E.C., Hutton M.L., Hentz J.G., Hoffman-Snyder C., Woodruff B.K., Alexander G.E., Osborne D. (2007). Cognitive Domain Decline in Healthy Apolipoprotein E Ε4 Homozygotes Before the Diagnosis of Mild Cognitive Impairment. Arch. Neurol..

[48] Li W., Qiu Q., Sun L., Li X., Xiao S. (2019). Short-Term Adverse Effects of the Apolipoprotein E Ε4 Allele over Language Function and Executive Function in Healthy Older Adults. NDT.

[49] Tensaouti Y., Stephanz E.P., Yu T.-S., Kernie S.G. (2018). ApoE Regulates the Development of Adult Newborn Hippocampal Neurons. eNeuro.

[50] Khalil Y.A., Rabès J.-P., Boileau C., Varret M. (2021). APOE Gene Variants in Primary Dyslipidemia. Atherosclerosis.

[51] Verghese P.B., Castellano J.M., Holtzman D.M. (2011). Apolipoprotein E in Alzheimer’s Disease and Other Neurological Disorders. Lancet Neurol..

[52] Federoff M., Jimenez-Rolando B., Nalls M.A., Singleton A.B. (2012). A Large Study Reveals No Association between APOE and Parkinson’s Disease. Neurobiol. Dis..

[53] Pu J., Jin C., Wang Z., Fang Y., Li Y., Xue N., Zheng R., Lin Z., Yan Y., Si X. (2022). Apolipoprotein E Genotype Contributes to Motor Progression in Parkinson’s Disease. Mov. Disord..

[54] Harrington C.R., Louwagie J., Rossau R., Vanmechelen E., Perry R.H., Perry E.K., Xuereb J.H., Roth M., Wischik C.M. (1994). Influence of Apolipoprotein E Genotype on Senile Dementia of the Alzheimer and Lewy Body Types. Significance for Etiological Theories of Alzheimer’s Disease. Am. J. Pathol..

[55] Raman S., Brookhouser N., Brafman D.A. (2020). Using Human Induced Pluripotent Stem Cells (HiPSCs) to Investigate the Mechanisms by Which Apolipoprotein E (APOE) Contributes to Alzheimer’s Disease (AD) Risk. Neurobiol. Dis..

[56] LaDu M.J., Falduto M.T., Manelli A.M., Reardon C.A., Getz G.S., Frail D.E. (1994). Isoform-Specific Binding of Apolipoprotein E to Beta-Amyloid. J. Biol. Chem..

[57] Lee C.Y.D., Landreth G.E. (2010). The Role of Microglia in Amyloid Clearance from the AD Brain. J. Neural. Transm..

[58] Deane R., Sagare A., Hamm K., Parisi M., Lane S., Finn M.B., Holtzman D.M., Zlokovic B.V. (2008). ApoE Isoform-Specific Disruption of Amyloid Beta Peptide Clearance from Mouse Brain. J. Clin. Investig..

[59] Verghese P.B., Castellano J.M., Garai K., Wang Y., Jiang H., Shah A., Bu G., Frieden C., Holtzman D.M. (2013). ApoE Influences Amyloid-β (Aβ) Clearance despite Minimal ApoE/Aβ Association in Physiological Conditions. Proc. Natl. Acad. Sci. USA.

[60] Jiang Q., Lee C.Y.D., Mandrekar S., Wilkinson B., Cramer P., Zelcer N., Mann K., Lamb B., Willson T.M., Collins J.L. (2008). ApoE Promotes the Proteolytic Degradation of Abeta. Neuron.

[61] Wang C., Xiong M., Gratuze M., Bao X., Shi Y., Andhey P.S., Manis M., Schroeder C., Yin Z., Madore C. (2021). Selective Removal of Astrocytic APOE4 Strongly Protects against Tau-Mediated Neurodegeneration and Decreases Synaptic Phagocytosis by Microglia. Neuron.

[62] Hamanaka H., Katoh-Fukui Y., Suzuki K., Kobayashi M., Suzuki R., Motegi Y., Nakahara Y., Takeshita A., Kawai M., Ishiguro K. (2000). Altered Cholesterol Metabolism in Human Apolipoprotein E4 Knock-in Mice. Hum. Mol. Genet..

[63] Har-Paz I., Arieli E., Moran A. (2021). ApoE4 Attenuates Cortical Neuronal Activity in Young Behaving ApoE4 Rats. Neurobiol. Dis..

[64] Har-Paz I., Roisman N., Michaelson D.M., Moran A. (2019). Extra-Hippocampal Learning Deficits in Young Apolipoprotein E4 Mice and Their Synaptic Underpinning. JAD.

[65] Lanfranco M.F., Sepulveda J., Kopetsky G., Rebeck G.W. (2021). Expression and Secretion of apoE Isoforms in Astrocytes and Microglia during Inflammation. Glia.

[66] Iannucci J., Sen A., Grammas P. (2021). Isoform-Specific Effects of Apolipoprotein E on Markers of Inflammation and Toxicity in Brain Glia and Neuronal Cells In Vitro. CIMB.

[67] Nguyen H.C., Qadura M., Singh K.K. (2020). Role of the Fatty Acid Binding Proteins in Cardiovascular Diseases: A Systematic Review. J. Clin. Med..

[68] Liu R.-Z., Mita R., Beaulieu M., Gao Z., Godbout R. (2010). Fatty Acid Binding Proteins in Brain Development and Disease. Int. J. Dev. Biol..

[69] Veerkamp J.H., Zimmerman A.W. (2001). Fatty Acid-Binding Proteins of Nervous Tissue. JMN.

[70] Islam A., Kagawa Y., Sharifi K., Ebrahimi M., Miyazaki H., Yasumoto Y., Kawamura S., Yamamoto Y., Sakaguti S., Sawada T. (2014). Fatty Acid Binding Protein 3 Is Involved in n–3 and n–6 PUFA Transport in Mouse Trophoblasts. J. Nutr..

[71] Oizumi H., Yamasaki K., Suzuki H., Hasegawa T., Sugimura Y., Baba T., Fukunaga K., Takeda A. (2021). Fatty Acid-Binding Protein 3 Expression in the Brain and Skin in Human Synucleinopathies. Front. Aging Neurosci..

[72] Shibasaki Y., Baillie D.A., St Clair D., Brookes A.J. (1995). High-Resolution Mapping of SNCA Encoding Alpha-Synuclein, the Non-A Beta Component of Alzheimer’s Disease Amyloid Precursor, to Human Chromosome 4q21.3-->q22 by Fluorescence in Situ Hybridization. Cytogenet. Cell Genet..

[73] Lashuel H.A., Overk C.R., Oueslati A., Masliah E. (2013). The Many Faces of α-Synuclein: From Structure and Toxicity to Therapeutic Target. Nat. Rev. Neurosci..

[74] Daniele S., Costa B., Pietrobono D., Giacomelli C., Iofrida C., Trincavelli M.L., Fusi J., Franzoni F., Martini C. (2018). Epigenetic Modifications of the α-Synuclein Gene and Relative Protein Content Are Affected by Ageing and Physical Exercise in Blood from Healthy Subjects. Oxid Med. Cell. Longev..

[75] Villar-Piqué A., Lopes da Fonseca T., Outeiro T.F. (2016). Structure, Function and Toxicity of Alpha-Synuclein: The Bermuda Triangle in Synucleinopathies. J. Neurochem..

[76] Vargas K.J., Makani S., Davis T., Westphal C.H., Castillo P.E., Chandra S.S. (2014). Synucleins Regulate the Kinetics of Synaptic Vesicle Endocytosis. J Neurosci..

[77] Matsuo K., Kawahata I., Melki R., Bousset L., Owada Y., Fukunaga K. (2021). Suppression of α-Synuclein Propagation after Intrastriatal Injection in FABP3 Null Mice. Brain Res..

[78] Kawahata I., Fukunaga K. (2022). Impact of Fatty Acid-Binding Proteins and Dopamine Receptors on α-Synucleinopathy. J. Pharmacol. Sci..

[79] Pacheco C.R., Morales C.N., Ramírez A.E., Muñoz F.J., Gallegos S.S., Caviedes P.A., Aguayo L.G., Opazo C.M. (2015). Extracellular α-Synuclein Alters Synaptic Transmission in Brain Neurons by Perforating the Neuronal Plasma Membrane. J. Neurochem..

[80] Chen L., Jin J., Davis J., Zhou Y., Wang Y., Liu J., Lockhart P.J., Zhang J. (2007). Oligomeric Alpha-Synuclein Inhibits Tubulin Polymerization. Biochem. Biophys. Res. Commun..

[81] Kröger H., Donner I., Skiello G. (1975). Influence of a New Virostatic Compound on the Induction of Enzymes in Rat Liver. Arzneimittelforschung.

[82] Colla E., Coune P., Liu Y., Pletnikova O., Troncoso J.C., Iwatsubo T., Schneider B.L., Lee M.K. (2012). Endoplasmic Reticulum Stress Is Important for the Manifestations of α-Synucleinopathy in Vivo. J. Neurosci..

[83] Klegeris A., Pelech S., Giasson B.I., Maguire J., Zhang H., McGeer E.G., McGeer P.L. (2008). α-Synuclein Activates Stress Signaling Protein Kinases in THP-1 Cells and Microglia. Neurobiol. Aging.

[84] Wang G., Pan J., Chen S.-D. (2012). Kinases and Kinase Signaling Pathways: Potential Therapeutic Targets in Parkinson’s Disease. Prog. Neurobiol..

[85] Alvarez-Erviti L., Couch Y., Richardson J., Cooper J.M., Wood M.J.A. (2011). Alpha-Synuclein Release by Neurons Activates the Inflammatory Response in a Microglial Cell Line. Neurosci. Res..

[86] Brosseron F., Kleemann K., Kolbe C., Santarelli F., Castro-Gomez S., Tacik P., Latz E., Jessen F., Heneka M.T. (2021). Interrelations of Alzheimer´s Disease Candidate Biomarkers Neurogranin, Fatty Acid-binding Protein 3 and Ferritin to Neurodegeneration and Neuroinflammation. J. Neurochem..

[87] Dulewicz M., Kulczyńska-Przybik A., Słowik A., Borawska R., Mroczko B. (2021). Fatty Acid Binding Protein 3 (FABP3) and Apolipoprotein E4 (ApoE4) as Lipid Metabolism-Related Biomarkers of Alzheimer’s Disease. JCM.

[88] Sepe F.N., Chiasserini D., Parnetti L. (2018). Role of FABP3 as Biomarker in Alzheimer’s Disease and Synucleinopathies. Future Neurol..

[89] Marion M., Hamilton J., Richardson B., Roeder N., Figueiredo A., Nubelo A., Hetelekides E., Penman S., Owada Y., Kagawa Y. (2022). Environmental Enrichment Sex-Dependently Rescues Memory Impairment in FABP5 KO Mice Not Mediated by Brain-Derived Neurotrophic Factor. Behav. Brain Res..

[90] Pan Y., Scanlon M.J., Owada Y., Yamamoto Y., Porter C.J.H., Nicolazzo J.A. (2015). Fatty Acid-Binding Protein 5 Facilitates the Blood–Brain Barrier Transport of Docosahexaenoic Acid. Mol. Pharm..

[91] Lauritzen L., Brambilla P., Mazzocchi A., Harsløf L., Ciappolino V., Agostoni C. (2016). DHA Effects in Brain Development and Function. Nutrients.

[92] Wang Y., Shinoda Y., Cheng A., Kawahata I., Fukunaga K. (2021). Epidermal Fatty Acid-Binding Protein 5 (FABP5) Involvement in Alpha-Synuclein-Induced Mitochondrial Injury under Oxidative Stress. Biomedicines.

[93] Macdonald R., Barnes K., Hastings C., Mortiboys H. (2018). Mitochondrial Abnormalities in Parkinson’s Disease and Alzheimer’s Disease: Can Mitochondria Be Targeted Therapeutically?. Biochem. Soc. Trans..

[94] Vicario M., Cieri D., Brini M., Calì T. (2018). The Close Encounter Between Alpha-Synuclein and Mitochondria. Front. Neurosci..

[95] Calì T., Ottolini D., Negro A., Brini M. (2012). α-Synuclein Controls Mitochondrial Calcium Homeostasis by Enhancing Endoplasmic Reticulum-Mitochondria Interactions. J. Biol. Chem..

[96] Pozo Devoto V.M., Dimopoulos N., Alloatti M., Pardi M.B., Saez T.M., Otero M.G., Cromberg L.E., Marín-Burgin A., Scassa M.E., Stokin G.B. (2017). αSynuclein Control of Mitochondrial Homeostasis in Human-Derived Neurons Is Disrupted by Mutations Associated with Parkinson’s Disease. Sci. Rep..

[97] Menges S., Minakaki G., Schaefer P.M., Meixner H., Prots I., Schlötzer-Schrehardt U., Friedland K., Winner B., Outeiro T.F., Winklhofer K.F. (2017). Alpha-Synuclein Prevents the Formation of Spherical Mitochondria and Apoptosis under Oxidative Stress. Sci. Rep..

[98] Gui Y.-X., Wang X.-Y., Kang W.-Y., Zhang Y.-J., Zhang Y., Zhou Y., Quinn T.J., Liu J., Chen S.-D. (2012). Extracellular Signal-Regulated Kinase Is Involved in Alpha-Synuclein-Induced Mitochondrial Dynamic Disorders by Regulating Dynamin-like Protein 1. Neurobiol. Aging.

[99] Devi L., Raghavendran V., Prabhu B.M., Avadhani N.G., Anandatheerthavarada H.K. (2008). Mitochondrial Import and Accumulation of α-Synuclein Impair Complex I in Human Dopaminergic Neuronal Cultures and Parkinson Disease Brain. J. Biol. Chem..

[100] Wang Y., Wahafu A., Wu W., Xiang J., Huo L., Ma X., Wang N., Liu H., Bai X., Xu D. (2021). FABP5 Enhances Malignancies of Lower-grade Gliomas via Canonical Activation of NF-κB Signaling. J. Cell. Mol. Med..

[101] Kipp M., Clarner T., Gingele S., Pott F., Amor S., van der Valk P., Beyer C. (2011). Brain Lipid Binding Protein (FABP7) as Modulator of Astrocyte Function. Physiol. Res..

[102] Ebrahimi M., Yamamoto Y., Sharifi K., Kida H., Kagawa Y., Yasumoto Y., Islam A., Miyazaki H., Shimamoto C., Maekawa M. (2016). Astrocyte-Expressed FABP7 Regulates Dendritic Morphology and Excitatory Synaptic Function of Cortical Neurons: Astrocyte FABP7 as a Regulator of Neuronal Morphology. Glia.

[103] Killoy K.M., Harlan B.A., Pehar M., Vargas M.R. (2020). FABP7 Upregulation Induces a Neurotoxic Phenotype in Astrocytes. Glia.

[104] Elsherbiny M.E., Emara M., Godbout R. (2013). Interaction of Brain Fatty Acid-Binding Protein with the Polyunsaturated Fatty Acid Environment as a Potential Determinant of Poor Prognosis in Malignant Glioma. Prog. Lipid Res..

[105] Asaro A., Sinha R., Bakun M., Kalnytska O., Carlo-Spiewok A.-S., Rubel T., Rozeboom A., Dadlez M., Kaminska B., Aronica E. (2021). ApoE4 Disrupts Interaction of Sortilin with Fatty Acid-Binding Protein 7 Essential to Promote Lipid Signaling. J. Cell. Sci..

[106] Duffy C.M., Xu H., Nixon J.P., Bernlohr D.A., Butterick T.A. (2017). Identification of a Fatty Acid Binding Protein4-UCP2 Axis Regulating Microglial Mediated Neuroinflammation. Mol. Cell. Neurosci..

[107] McFarlane O., Kędziora-Kornatowska K. (2020). Cholesterol and Dementia: A Long and Complicated Relationship. CAS.

[108] Noguchi N., Saito Y., Urano Y. (2014). Diverse Functions of 24(S)-Hydroxycholesterol in the Brain. Biochem. Biophys. Res. Commun..

[109] Goedeke L., Fernández-Hernando C. (2014). MicroRNAs: A Connection between Cholesterol Metabolism and Neurodegeneration. Neurobiol. Dis..

[110] Gamba P., Testa G., Gargiulo S., Staurenghi E., Poli G., Leonarduzzi G. (2015). Oxidized Cholesterol as the Driving Force behind the Development of Alzheimer’s Disease. Front. Aging Neurosci..

[111] George K.S., Wu S. (2012). Lipid Raft: A Floating Island of Death or Survival. Toxicol. Appl. Pharmacol..

[112] García-Sanz P., Aerts J., Moratalla R. (2021). The Role of Cholesterol in α-Synuclein and Lewy Body Pathology in *GBA1* Parkinson’s Disease. Mov. Disord..

[113] García-Sanz P., Orgaz L., Bueno-Gil G., Espadas I., Rodríguez-Traver E., Kulisevsky J., Gutierrez A., Dávila J.C., González-Polo R.A., Fuentes J.M. (2017). N370S *-GBA1* Mutation Causes Lysosomal Cholesterol Accumulation in Parkinson’s Disease: Cholesterol Accumulates in *GBA1* -PD Lysosomes. Mov. Disord..

[114] García-Sanz P., Orgaz L., Fuentes J.M., Vicario C., Moratalla R. (2018). Cholesterol and Multilamellar Bodies: Lysosomal Dysfunction in *GBA* -Parkinson Disease. Autophagy.

[115] Snezhkina A.V., Kudryavtseva A.V., Kardymon O.L., Savvateeva M.V., Melnikova N.V., Krasnov G.S., Dmitriev A.A. (2019). ROS Generation and Antioxidant Defense Systems in Normal and Malignant Cells. Oxid. Med. Cell. Longev..

[116] Schieber M., Chandel N.S. (2014). ROS Function in Redox Signaling and Oxidative Stress. Curr. Biol..

[117] Oktyabrsky O.N., Smirnova G.V. (2007). Redox Regulation of Cellular Functions. Biochemistry.

[118] Lennicke C., Cochemé H.M. (2021). Redox Metabolism: ROS as Specific Molecular Regulators of Cell Signaling and Function. Mol. Cell.

[119] Sultana R., Perluigi M., Butterfield D.A. (2013). Lipid Peroxidation Triggers Neurodegeneration: A Redox Proteomics View into the Alzheimer Disease Brain. Free Radic. Biol. Med..

[120] Angelova P.R., Esteras N., Abramov A.Y. (2021). Mitochondria and Lipid Peroxidation in the Mechanism of Neurodegeneration: Finding Ways for Prevention. Med. Res. Rev..

[121] Angelova P.R., Abramov A.Y. (2016). Functional Role of Mitochondrial Reactive Oxygen Species in Physiology. Free Radic. Biol. Med..

[122] Abramov A.Y., Scorziello A., Duchen M.R. (2007). Three Distinct Mechanisms Generate Oxygen Free Radicals in Neurons and Contribute to Cell Death during Anoxia and Reoxygenation. J. Neurosci..

[123] Gandhi S., Abramov A.Y. (2012). Mechanism of Oxidative Stress in Neurodegeneration. Oxid. Med. Cell. Longev..

[124] Shichiri M. (2014). The Role of Lipid Peroxidation in Neurological Disorders. J. Clin. Biochem. Nutr..

[125] Petrovic S., Arsic A., Ristic-Medic D., Cvetkovic Z., Vucic V. (2020). Lipid Peroxidation and Antioxidant Supplementation in Neurodegenerative Diseases: A Review of Human Studies. Antioxidants.

[126] Tadokoro K., Ohta Y., Inufusa H., Loon A.F.N., Abe K. (2020). Prevention of Cognitive Decline in Alzheimer’s Disease by Novel Antioxidative Supplements. IJMS.

[127] Butterfield D.A., Mattson M.P. (2020). Apolipoprotein E and Oxidative Stress in Brain with Relevance to Alzheimer’s Disease. Neurobiol. Dis..

[128] Farooqui A.A., Horrocks L.A. (1998). Lipid Peroxides in the Free Radical Pathophysiology of Brain Diseases. Cell. Mol. Neurobiol..

[129] Anzai K., Ogawa K., Goto Y., Senzaki Y., Ozawa T., Yamamoto H. (1999). Oxidation-Dependent Changes in the Stability and Permeability of Lipid Bilayers. Antioxid Redox Signal..

[130] Yehuda S., Rabinovitz S., Carasso R.L., Mostofsky D.I. (2002). The Role of Polyunsaturated Fatty Acids in Restoring the Aging Neuronal Membrane. Neurobiol. Aging.

[131] Moreira P.I., Santos M.S., Oliveira C.R., Shenk J.C., Nunomura A., Smith M.A., Zhu X., Perry G. (2008). Alzheimer Disease and the Role of Free Radicals in the Pathogenesis of the Disease. CNS Neurol. Disord. Drug Targets.

[132] Daniele S., Giacomelli C., Martini C. (2018). Brain Ageing and Neurodegenerative Disease: The Role of Cellular Waste Management. Biochem. Pharmacol..

[133] Giacomelli C., Daniele S., Martini C. (2017). Potential Biomarkers and Novel Pharmacological Targets in Protein Aggregation-Related Neurodegenerative Diseases. Biochem. Pharmacol..

[134] Butterfield D.A., Bader Lange M.L., Sultana R. (2010). Involvements of the Lipid Peroxidation Product, HNE, in the Pathogenesis and Progression of Alzheimer’s Disease. Biochim. Biophys. Acta.

[135] Lee J., Kosaras B., Del Signore S.J., Cormier K., McKee A., Ratan R.R., Kowall N.W., Ryu H. (2011). Modulation of Lipid Peroxidation and Mitochondrial Function Improves Neuropathology in Huntington’s Disease Mice. Acta Neuropathol..

[136] Ruipérez V., Darios F., Davletov B. (2010). Alpha-Synuclein, Lipids and Parkinson’s Disease. Prog. Lipid Res..

[137] Sajdel-Sulkowska E.M., Marotta C.A. (1984). Alzheimer’s Disease Brain: Alterations in RNA Levels and in a Ribonuclease-Inhibitor Complex. Science.

[138] Shichiri M., Yoshida Y., Ishida N., Hagihara Y., Iwahashi H., Tamai H., Niki E. (2011). α-Tocopherol Suppresses Lipid Peroxidation and Behavioral and Cognitive Impairments in the Ts65Dn Mouse Model of Down Syndrome. Free Radic. Biol. Med..

[139] Vaarmann A., Gandhi S., Abramov A.Y. (2010). Dopamine Induces Ca^2+^ Signaling in Astrocytes through Reactive Oxygen Species Generated by Monoamine Oxidase. J. Biol. Chem..

[140] Ademowo O.S., Dias H.K.I., Burton D.G.A., Griffiths H.R. (2017). Lipid (per) Oxidation in Mitochondria: An Emerging Target in the Ageing Process?. Biogerontology.

[141] Shen Z., Ye C., McCain K., Greenberg M.L. (2015). The Role of Cardiolipin in Cardiovascular Health. Biomed Res. Int..

[142] Pienaar I.S., Elson J.L., Racca C., Nelson G., Turnbull D.M., Morris C.M. (2013). Mitochondrial Abnormality Associates with Type-Specific Neuronal Loss and Cell Morphology Changes in the Pedunculopontine Nucleus in Parkinson Disease. Am. J. Pathol..

[143] Chandrasekaran K., Giordano T., Brady D.R., Stoll J., Martin L.J., Rapoport S.I. (1994). Impairment in Mitochondrial Cytochrome Oxidase Gene Expression in Alzheimer Disease. Mol. Brain Res..

[144] Chen L., Na R., Gu M., Richardson A., Ran Q. (2008). Lipid Peroxidation Up-Regulates BACE1 Expression *in Vivo*: A Possible Early Event of Amyloidogenesis in Alzheimer’s Disease. J. Neurochem..

[145] Tan B.L., Norhaizan M.E., Liew W.-P.-P., Sulaiman Rahman H. (2018). Antioxidant and Oxidative Stress: A Mutual Interplay in Age-Related Diseases. Front. Pharmacol..

[146] Oppedisano F., Maiuolo J., Gliozzi M., Musolino V., Carresi C., Nucera S., Scicchitano M., Scarano F., Bosco F., Macrì R. (2020). The Potential for Natural Antioxidant Supplementation in the Early Stages of Neurodegenerative Disorders. IJMS.

[147] Kontush A., Mann U., Arlt S., Ujeyl A., Lührs C., Müller-Thomsen T., Beisiegel U. (2001). Influence of Vitamin E and C Supplementation on Lipoprotein Oxidation in Patients with Alzheimer’s Disease. Free Radic. Biol. Med..

[148] Kiecolt-Glaser J.K., Epel E.S., Belury M.A., Andridge R., Lin J., Glaser R., Malarkey W.B., Hwang B.S., Blackburn E. (2013). Omega-3 Fatty Acids, Oxidative Stress, and Leukocyte Telomere Length: A Randomized Controlled Trial. Brain Behav. Immun..

[149] Mohammad N.S., Nazli R., Zafar H., Fatima S. (2022). Effects of Lipid Based Multiple Micronutrients Supplement on the Birth Outcome of Underweight Pre-Eclamptic Women: A Randomized Clinical Trial. Pak. J. Med. Sci..

[150] Moss J.W.E., Williams J.O., Ramji D.P. (2018). Nutraceuticals as Therapeutic Agents for Atherosclerosis. Biochim. Biophys. Acta Mol. Basis Dis..

[151] Ribeiro S.M.L., Luz S.D.S., Aquino R.d.C. (2015). The Role of Nutrition and Physical Activity in Cholesterol and Aging. Clin. Geriatr. Med..

[152] Demonty I., Ras R.T., van der Knaap H.C.M., Duchateau G.S.M.J.E., Meijer L., Zock P.L., Geleijnse J.M., Trautwein E.A. (2009). Continuous Dose-Response Relationship of the LDL-Cholesterol-Lowering Effect of Phytosterol Intake. J. Nutr..

[153] Kaur R., Myrie S.B. (2020). Association of Dietary Phytosterols with Cardiovascular Disease Biomarkers in Humans. Lipids.

[154] Mooradian A.D., Haas M.J. (2014). The Effect of Nutritional Supplements on Serum High-Density Lipoprotein Cholesterol and Apolipoprotein A-I. Am. J. Cardiovasc. Drugs.

[155] Xu Y., Nisenblat V., Lu C., Li R., Qiao J., Zhen X., Wang S. (2018). Pretreatment with Coenzyme Q10 Improves Ovarian Response and Embryo Quality in Low-Prognosis Young Women with Decreased Ovarian Reserve: A Randomized Controlled Trial. Reprod Biol. Endocrinol..

[156] Sawaddiruk P., Apaijai N., Paiboonworachat S., Kaewchur T., Kasitanon N., Jaiwongkam T., Kerdphoo S., Chattipakorn N., Chattipakorn S.C. (2019). Coenzyme Q10 Supplementation Alleviates Pain in Pregabalin-Treated Fibromyalgia Patients *via* Reducing Brain Activity and Mitochondrial Dysfunction. Free Radic. Res..

[157] Mischley L.K., Allen J., Bradley R. (2012). Coenzyme Q10 Deficiency in Patients with Parkinson’s Disease. J. Neurol. Sci..

[158] Moussa C., Hebron M., Huang X., Ahn J., Rissman R.A., Aisen P.S., Turner R.S. (2017). Resveratrol Regulates Neuro-Inflammation and Induces Adaptive Immunity in Alzheimer’s Disease. J. Neuroinflam..

[159] Bo S., Togliatto G., Gambino R., Ponzo V., Lombardo G., Rosato R., Cassader M., Brizzi M.F. (2018). Impact of Sirtuin-1 Expression on H3K56 Acetylation and Oxidative Stress: A Double-Blind Randomized Controlled Trial with Resveratrol Supplementation. Acta Diabetol..

[160] Ochiai R., Saitou K., Suzukamo C., Osaki N., Asada T. (2019). Effect of Chlorogenic Acids on Cognitive Function in Mild Cognitive Impairment: A Randomized Controlled Crossover Trial. JAD.

[161] Oboh G., Agunloye O.M., Akinyemi A.J., Ademiluyi A.O., Adefegha S.A. (2013). Comparative Study on the Inhibitory Effect of Caffeic and Chlorogenic Acids on Key Enzymes Linked to Alzheimer’s Disease and Some Pro-Oxidant Induced Oxidative Stress in Rats’ Brain-In Vitro. Neurochem. Res..

[162] Samarghandian S., Azimi-Nezhad M., Farkhondeh T., Samini F. (2017). Anti-Oxidative Effects of Curcumin on Immobilization-Induced Oxidative Stress in Rat Brain, Liver and Kidney. Biomed. Pharmacother..

[163] Taïlé J., Arcambal A., Clerc P., Gauvin-Bialecki A., Gonthier M.-P. (2020). Medicinal Plant Polyphenols Attenuate Oxidative Stress and Improve Inflammatory and Vasoactive Markers in Cerebral Endothelial Cells during Hyperglycemic Condition. Antioxidants.

[164] Cicero A.F.G., Colletti A. (2018). Polyphenols Effect on Circulating Lipids and Lipoproteins: From Biochemistry to Clinical Evidence. Curr. Pharm. Des..

[165] Castro-Barquero S., Tresserra-Rimbau A., Vitelli-Storelli F., Doménech M., Salas-Salvadó J., Martín-Sánchez V., Rubín-García M., Buil-Cosiales P., Corella D., Fitó M. (2020). Dietary Polyphenol Intake Is Associated with HDL-Cholesterol and A Better Profile of Other Components of the Metabolic Syndrome: A PREDIMED-Plus Sub-Study. Nutrients.

[166] Kapiotis S., Hermann M., Held I., Seelos C., Ehringer H., Gmeiner B.M. (1997). Genistein, the Dietary-Derived Angiogenesis Inhibitor, Prevents LDL Oxidation and Protects Endothelial Cells from Damage by Atherogenic LDL. Arter. Thromb. Vasc. Biol..

[167] Demir E., Taysi S., Ulusal H., Kaplan D.S., Cinar K., Tarakcioglu M. (2020). *Nigella Sativa* Oil and Thymoquinone Reduce Oxidative Stress in the Brain Tissue of Rats Exposed to Total Head Irradiation. Int. J. Radiat. Biol..

[168] Jafari F., Amani R., Tarrahi M.J. (2020). Effect of Zinc Supplementation on Physical and Psychological Symptoms, Biomarkers of Inflammation, Oxidative Stress, and Brain-Derived Neurotrophic Factor in Young Women with Premenstrual Syndrome: A Randomized, Double-Blind, Placebo-Controlled Trial. Biol. Trace Elem. Res..

[169] Ebokaiwe A.P., Okori S., Nwankwo J.O., Ejike C.E.C.C., Osawe S.O. (2021). Selenium Nanoparticles and Metformin Ameliorate Streptozotocin-Instigated Brain Oxidative-Inflammatory Stress and Neurobehavioral Alterations in Rats. Naunyn-Schmiedeberg’s Arch. Pharmacol..

[170] Wu J.Y., Reaves S.K., Wang Y.R., Wu Y., Lei P.P., Lei K.Y. (1998). Zinc Deficiency Decreases Plasma Level and Hepatic MRNA Abundance of Apolipoprotein A-I in Rats and Hamsters. Am. J. Physiol..

[171] Cheng L.-H., Liu Y.-W., Wu C.-C., Wang S., Tsai Y.-C. (2019). Psychobiotics in Mental Health, Neurodegenerative and Neurodevelopmental Disorders. J. Food Drug Anal..

[172] Athari Nik Azm S., Djazayeri A., Safa M., Azami K., Ahmadvand B., Sabbaghziarani F., Sharifzadeh M., Vafa M. (2018). Lactobacilli and Bifidobacteria Ameliorate Memory and Learning Deficits and Oxidative Stress in β-Amyloid (1–42) Injected Rats. Appl. Physiol. Nutr. Metab..

[173] Musa N.H., Mani V., Lim S.M., Vidyadaran S., Abdul Majeed A.B., Ramasamy K. (2017). Lactobacilli-Fermented Cow’s Milk Attenuated Lipopolysaccharide-Induced Neuroinflammation and Memory Impairment in Vitro and in Vivo. J. Dairy Res..

[174] Akbari E., Asemi Z., Daneshvar Kakhaki R., Bahmani F., Kouchaki E., Tamtaji O.R., Hamidi G.A., Salami M. (2016). Effect of Probiotic Supplementation on Cognitive Function and Metabolic Status in Alzheimer’s Disease: A Randomized, Double-Blind and Controlled Trial. Front. Aging Neurosci..

[175] Bonfili L., Cecarini V., Cuccioloni M., Angeletti M., Berardi S., Scarpona S., Rossi G., Eleuteri A.M. (2018). SLAB51 Probiotic Formulation Activates SIRT1 Pathway Promoting Antioxidant and Neuroprotective Effects in an AD Mouse Model. Mol. Neurobiol..

[176] Kim D., Nguyen M.D., Dobbin M.M., Fischer A., Sananbenesi F., Rodgers J.T., Delalle I., Baur J.A., Sui G., Armour S.M. (2007). SIRT1 Deacetylase Protects against Neurodegeneration in Models for Alzheimer’s Disease and Amyotrophic Lateral Sclerosis. EMBO J..

[177] Tamtaji O.R., Taghizadeh M., Daneshvar Kakhaki R., Kouchaki E., Bahmani F., Borzabadi S., Oryan S., Mafi A., Asemi Z. (2019). Clinical and Metabolic Response to Probiotic Administration in People with Parkinson’s Disease: A Randomized, Double-Blind, Placebo-Controlled Trial. Clin. Nutr..

[178] Borzabadi S., Oryan S., Eidi A., Aghadavod E., Daneshvar Kakhaki R., Tamtaji O.R., Taghizadeh M., Asemi Z. (2018). The Effects of Probiotic Supplementation on Gene Expression Related to Inflammation, Insulin and Lipid in Patients with Parkinson’s Disease: A Randomized, Double-Blind, PlaceboControlled Trial. Arch. Iran. Med..

[179] Liu Y., Yan T., Chu J.M.-T., Chen Y., Dunnett S., Ho Y.-S., Wong G.T.-C., Chang R.C.-C. (2019). The Beneficial Effects of Physical Exercise in the Brain and Related Pathophysiological Mechanisms in Neurodegenerative Diseases. Lab. Investig..

[180] Erickson K.I., Voss M.W., Prakash R.S., Basak C., Szabo A., Chaddock L., Kim J.S., Heo S., Alves H., White S.M. (2011). Exercise Training Increases Size of Hippocampus and Improves Memory. Proc. Natl. Acad. Sci. USA.

[181] Won J., Callow D.D., Pena G.S., Jordan L.S., Arnold-Nedimala N.A., Nielson K.A., Smith J.C. (2021). Hippocampal Functional Connectivity and Memory Performance After Exercise Intervention in Older Adults with Mild Cognitive Impairment. JAD.

[182] Arazi H., Babaei P., Moghimi M., Asadi A. (2021). Acute Effects of Strength and Endurance Exercise on Serum BDNF and IGF-1 Levels in Older Men. BMC Geriatr..

[183] Colucci-D’Amato L., Speranza L., Volpicelli F. (2020). Neurotrophic Factor BDNF, Physiological Functions and Therapeutic Potential in Depression, Neurodegeneration and Brain Cancer. IJMS.

[184] von Bohlen und Halbach O., von Bohlen und Halbach V. (2018). BDNF Effects on Dendritic Spine Morphology and Hippocampal Function. Cell Tissue Res..

[185] Lin J.-Y., Kuo W.-W., Baskaran R., Kuo C.-H., Chen Y.-A., Chen W.S.-T., Ho T.-J., Day C.H., Mahalakshmi B., Huang C.-Y. (2020). Swimming Exercise Stimulates IGF1/ PI3K/Akt and AMPK/SIRT1/PGC1α Survival Signaling to Suppress Apoptosis and Inflammation in Aging Hippocampus. Aging.

[186] Scisciola L., Fontanella R.A., Surina, Cataldo V., Paolisso G., Barbieri M. (2021). Sarcopenia and Cognitive Function: Role of Myokines in Muscle Brain Cross-Talk. Life.

[187] Wrann C.D., White J.P., Salogiannnis J., Laznik-Bogoslavski D., Wu J., Ma D., Lin J.D., Greenberg M.E., Spiegelman B.M. (2013). Exercise Induces Hippocampal BDNF through a PGC-1α/FNDC5 Pathway. Cell Metab..

[188] Lourenco M.V., Frozza R.L., de Freitas G.B., Zhang H., Kincheski G.C., Ribeiro F.C., Gonçalves R.A., Clarke J.R., Beckman D., Staniszewski A. (2019). Exercise-Linked FNDC5/Irisin Rescues Synaptic Plasticity and Memory Defects in Alzheimer’s Models. Nat. Med..

[189] Peng J., Deng X., Huang W., Yu J., Wang J., Wang J., Yang S., Liu X., Wang L., Zhang Y. (2017). Irisin Protects against Neuronal Injury Induced by Oxygen-Glucose Deprivation in Part Depends on the Inhibition of ROS-NLRP3 Inflammatory Signaling Pathway. Mol. Immunol..

[190] Abd El-Kader S.M., Al-Jiffri O.H. (2019). Aerobic Exercise Modulates Cytokine Profile and Sleep Quality in Elderly. Afr. Health Sci..

[191] Abd El-Kader S.M., Al-Shreef F.M. (2018). Inflammatory Cytokines and Immune System Modulation by Aerobic versus Resisted Exercise Training for Elderly. Afr. Health Sci..

[192] Abd El-Kader S.M., Al-Shreef F.M., Al-Jiffri O.H. (1970). Impact of Aerobic Exercise versus Resisted Exercise on Endothelial Activation Markers and Inflammatory Cytokines among Elderly. Afr. Health Sci..

[193] Mee-Inta O., Zhao Z.-W., Kuo Y.-M. (2019). Physical Exercise Inhibits Inflammation and Microglial Activation. Cells.

[194] (2017). Retracted: Treadmill Training Increases SIRT-1 and PGC-1 *α* Protein Levels and AMPK Phosphorylation in Quadriceps of Middle-Aged Rats in an Intensity-Dependent Manner. Mediat. Inflamm..

[195] Lu Y., Dong Y., Tucker D., Wang R., Ahmed M.E., Brann D., Zhang Q. (2017). Treadmill Exercise Exerts Neuroprotection and Regulates Microglial Polarization and Oxidative Stress in a Streptozotocin-Induced Rat Model of Sporadic Alzheimer’s Disease. JAD.

[196] Boccatonda A., Tripaldi R., Davì G., Santilli F. (2016). Oxidative Stress Modulation Through Habitual Physical Activity. CPD.

[197] Pingitore A., Lima G.P.P., Mastorci F., Quinones A., Iervasi G., Vassalle C. (2015). Exercise and Oxidative Stress: Potential Effects of Antioxidant Dietary Strategies in Sports. Nutrition.

[198] Cammisuli D.M., Bonuccelli U., Daniele S., Martini C., Fusi J., Franzoni F. (2020). Aerobic Exercise and Healthy Nutrition as Neuroprotective Agents for Brain Health in Patients with Parkinson’s Disease: A Critical Review of the Literature. Antioxidants.

[199] Piccarducci R., Daniele S., Fusi J., Chico L., Baldacci F., Siciliano G., Bonuccelli U., Franzoni F., Martini C. (2019). Impact of ApoE Polymorphism and Physical Activity on Plasma Antioxidant Capability and Erythrocyte Membranes. Antioxidants.

[200] Nawaz A., Batool Z., Shazad S., Rafiq S., Afzal A., Haider S. (2018). Physical Enrichment Enhances Memory Function by Regulating Stress Hormone and Brain Acetylcholinesterase Activity in Rats Exposed to Restraint Stress. Life Sci..

[201] Haider S., Saleem S., Perveen T., Tabassum S., Batool Z., Sadir S., Liaquat L., Madiha S. (2014). Age-Related Learning and Memory Deficits in Rats: Role of Altered Brain Neurotransmitters, Acetylcholinesterase Activity and Changes in Antioxidant Defense System. AGE.

[202] Pekny M., Wilhelmsson U., Pekna M. (2014). The Dual Role of Astrocyte Activation and Reactive Gliosis. Neurosci. Lett..

[203] Belaya I., Ivanova M., Sorvari A., Ilicic M., Loppi S., Koivisto H., Varricchio A., Tikkanen H., Walker F.R., Atalay M. (2020). Astrocyte Remodeling in the Beneficial Effects of Long-Term Voluntary Exercise in Alzheimer’s Disease. J. Neuroinflammation.

[204] Leardini-Tristão M., Andrade G., Garcia C., Reis P.A., Lourenço M., Moreira E.T.S., Lima F.R.S., Castro-Faria-Neto H.C., Tibirica E., Estato V. (2020). Physical Exercise Promotes Astrocyte Coverage of Microvessels in a Model of Chronic Cerebral Hypoperfusion. J. Neuroinflammation.

[205] Shen K., Liu X., Chen D., Chang J., Zhang Y., Kou X. (2021). Voluntary Wheel-Running Exercise Attenuates Brain Aging of Rats through Activating MiR-130a-Mediated Autophagy. Brain Res. Bull..

[206] Kwon I., Jang Y., Lee Y. (2021). Endurance Exercise-Induced Autophagy/Mitophagy Coincides with a Reinforced Anabolic State and Increased Mitochondrial Turnover in the Cortex of Young Male Mouse Brain. J. Mol. Neurosci..

[207] Chen D., Zhang Y., Zhang M., Chang J., Zeng Z., Kou X., Chen N. (2020). Exercise Attenuates Brain Aging by Rescuing Down-Regulated Wnt/β-Catenin Signaling in Aged Rats. Front. Aging Neurosci..

[208] Ahlskog J.E. (2018). Aerobic Exercise: Evidence for a Direct Brain Effect to Slow Parkinson Disease Progression. Mayo Clin. Proc..

[209] Sacheli M.A., Neva J.L., Lakhani B., Murray D.K., Vafai N., Shahinfard E., English C., McCormick S., Dinelle K., Neilson N. (2019). Exercise Increases Caudate Dopamine Release and Ventral Striatal Activation in Parkinson’s Disease. Mov. Disord.

[210] Cicero A.F.G., Colletti A. (2018). An Update on the Safety of Nutraceuticals and Effects on Lipid Parameters. Expert Opin. Drug Saf..

[211] Espín J.C., García-Conesa M.T., Tomás-Barberán F.A. (2007). Nutraceuticals: Facts and Fiction. Phytochemistry.

[212] Singh R.B., Watson R.R., Takahashi T. (2019). The Role of Functional Food Security in Global Health.

[213] Das L., Bhaumik E., Raychaudhuri U., Chakraborty R. (2012). Role of Nutraceuticals in Human Health. J. Food Sci. Technol..

[214] Cencic A., Chingwaru W. (2010). The Role of Functional Foods, Nutraceuticals, and Food Supplements in Intestinal Health. Nutrients.

[215] Wang Y., Xu D. (2017). Effects of Aerobic Exercise on Lipids and Lipoproteins. Lipids Health Dis..

